# Cushing's Syndrome and Fetal Features Resurgence in Adrenal Cortex–Specific *Prkar1a* Knockout Mice

**DOI:** 10.1371/journal.pgen.1000980

**Published:** 2010-06-10

**Authors:** Isabelle Sahut-Barnola, Cyrille de Joussineau, Pierre Val, Sarah Lambert-Langlais, Christelle Damon, Anne-Marie Lefrançois-Martinez, Jean-Christophe Pointud, Geoffroy Marceau, Vincent Sapin, Frédérique Tissier, Bruno Ragazzon, Jérôme Bertherat, Lawrence S. Kirschner, Constantine A. Stratakis, Antoine Martinez

**Affiliations:** 1 CNRS UMR6247, Génétique Reproduction et Développement (GReD), Clermont Université, Aubière, France; 2 Laboratoire de Biochimie, Centre de Biologie, CHU G. Montpied, Clermont-Ferrand, France; 3 INSERM U567, CNRS UMR8104, Institut Cochin, Department of Endocrinologie, Métabolisme, et Cancer, Université Paris Descartes, AP-HP Hôpital Cochin, France; 4 Department of Molecular Virology, Immunology, and Medical Genetics, Ohio State University, Columbus, Ohio, United States of America; 5 Division of Endocrinology, Diabetes, and Metabolism, Department of Internal Medicine, Ohio State University, Columbus, Ohio, United States of America; 6 Section on Endocrinology and Genetics, Program on Developmental Endocrinology and Genetics, Eunice Kennedy Shriver National Institute of Child Health and Human Development, NIH, Bethesda, Maryland, United States of America; University of Washington, United States of America

## Abstract

Carney complex (CNC) is an inherited neoplasia syndrome with endocrine overactivity. Its most frequent endocrine manifestation is primary pigmented nodular adrenocortical disease (PPNAD), a bilateral adrenocortical hyperplasia causing pituitary-independent Cushing's syndrome. Inactivating mutations in *PRKAR1A*, a gene encoding the type 1 α-regulatory subunit (R1α) of the cAMP–dependent protein kinase (PKA) have been found in 80% of CNC patients with Cushing's syndrome. To demonstrate the implication of R1α loss in the initiation and development of PPNAD, we generated mice lacking *Prkar1a* specifically in the adrenal cortex (AdKO). AdKO mice develop pituitary-independent Cushing's syndrome with increased PKA activity. This leads to autonomous steroidogenic genes expression and deregulated adreno-cortical cells differentiation, increased proliferation and resistance to apoptosis. Unexpectedly, R1α loss results in improper maintenance and centrifugal expansion of cortisol-producing fetal adrenocortical cells with concomitant regression of adult cortex. Our data provide the first *in vivo* evidence that loss of R1α is sufficient to induce autonomous adrenal hyper-activity and bilateral hyperplasia, both observed in human PPNAD. Furthermore, this model demonstrates that deregulated PKA activity favors the emergence of a new cell population potentially arising from the fetal adrenal, giving new insight into the mechanisms leading to PPNAD.

## Author Summary

Carney complex is a rare familial disease characterized by a predisposition to develop multiple endocrine tumors and highly morbid syndromes due to endocrine overactivities. Its most frequent endocrine manifestation, hypersecretion of glucocorticoids *i.e.* Cushing's syndrome, is caused by micronodular adrenal gland hyperplasia, an unusual neoplasia which combines both hyperplastic and atrophic areas. Inactivating mutations of the gene encoding the regulatory subunit 1α (R1α) of the cAMP–dependent protein kinase were frequently found in these patients, but the causal link between loss of R1α and onset of this adrenal disorder had not yet been established. Here, we describe the first mouse model mimicking this disease and provide mechanistic insights into endocrine overactivity and neoplastic transformation. Indeed, we show that lack of R1α induces autonomous expression of genes involved in steroid biosynthesis and resurgence of hyperplastic fetal-like cells with concomitant defects in cell renewal of the adult cortex. Our data therefore represent a substantial conceptual advance on the cellular dynamics involved in adrenal gland homeostasis. They suggest that regression of fetal structures may be important to establish normal endocrine functions and to allow cell renewal in the definitive cortex. Failure to clear out cells of fetal features in R1α-deficient adrenals leads to morbid hyperplasia.

## Introduction

Primary pigmented nodular adrenocortical disease (PPNAD) is a rare form of bilateral micronodular adrenocortical hyperplasia leading to high morbidity due to ACTH (adreno corticotropic hormone)-independent Cushing's syndrome. PPNAD may be either sporadic or regarded as the most frequent endocrine manifestation of Carney complex (CNC), an autosomal dominant multiple neoplasia syndrome characterized by cardiac myxomas, spotty skin pigmentation and endocrine overactivity [1]. Cushing's syndrome in PPNAD is most diagnosed in children and young adults. Both isolated PPNAD and CNC have been associated with inactivating mutations in *PRKAR1A*, the gene encoding the type 1α regulatory subunit (R1α) of the cAMP-dependent protein kinase (PKA) [2], [3]. Among CNC patients with Cushing's syndrome, the frequency of *PRKAR1A* mutations is about 80%. Tumour-specific loss of heterozygosity within the chromosomal region harboring *PRKAR1A* is observed in tumours from CNC patients and isolated PPNAD, suggesting that *PRKAR1A* is a potential tumour suppressor gene [4]. Because general homozygous loss of *Prkar1a* is lethal in early mouse embryos, various haploinsufficiency and tissue-specific knock-out models have been engineered to demonstrate its tumour suppressor activity [5], [6]. General down-regulation of R1α levels has been achieved either in mouse lines heterozygous for a null allele of *Prkar1a* [6], [7] or in a transgenic line carrying an inducible antisense-construct [8]. Both approaches indicate that haploinsufficiency for *Prkar1a* predisposes to tumour formation in a spectrum of endocrine and non-endocrine tissues that are cAMP-responsive; the mouse phenotype partially overlaps with the human one. However haploinsufficiency in mouse models does not appear to be sufficient to promote tumour formation in a subset of tissues known for their propensity to develop neoplasms in CNC patients. Thus, complete loss of *Prkar1a* using heart-, Schwann cell- or pituitary-specific knockouts was required to induce tumours in these tissues [9]–[11]. To date, although PPNAD is the most frequent endocrine disorder observed in CNC patients [12], little is known on its pathophysiology. No clear adrenal lesions nor Cushing's syndrome were observed in mouse models of haploinsufficiency, suggesting that complete loss of *Prkar1a* might be required to phenocopy human phenotype. To address directly this question and obtain a possible mouse model for PPNAD, we produced mice with targeted *Prkar1a* gene inactivation in adreno-cortical cells by mating *Prkar1a* floxed mice with *Akr1b7-*Cre mouse line, a Cre expressing line allowing specific gene ablation in the steroidogenic lineage of the adrenals [13]. Adrenal cortex-specific *Prkar1a* knockout mice (AdKO) develop pituitary-independent Cushing's syndrome and evident signs of deregulated adreno-cortical cells differentiation and hyperplasia. These defects lead to improper maintenance and expansion of foetal adrenal cells in adult adrenals and establishment of tumoural conditions. Deregulation of the inhibin-activin signalling pathway seems to be implicated in this improper maintenance in AdKO mice model and in the human pathology. Our data provide the first *in vivo* evidence that the absence of R1α subunit of PKA is sufficient to induce the autonomous adrenal hyper-activity and bilateral hyperplasia observed in PPNAD. They also strongly suggest that deregulated PKA activity positively affects the maintenance of foetal characteristics in adult adrenal glands.

## Results

### Efficient ablation of *Prkar1a* in the adrenal cortex of AdKO mice

**Figure 1:**
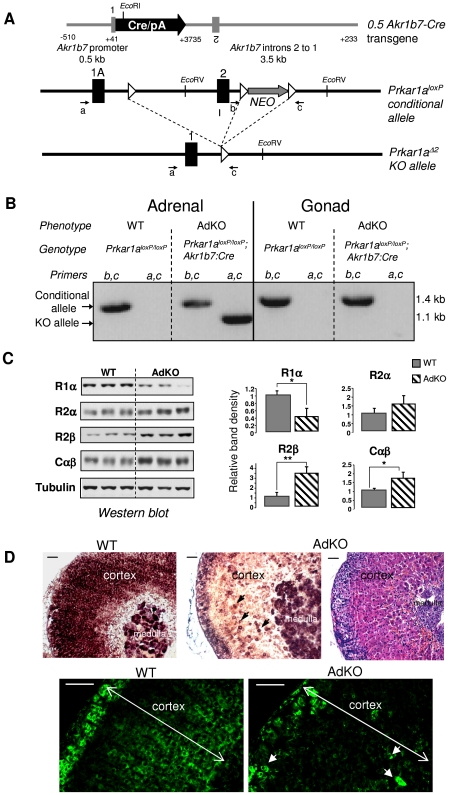
Generation of a conditional knockout for *Prkar1a* in mouse adrenal cortex (AdKO mice). (A) Scheme of the 0.5 *Akr1b7*-Cre transgene driving the Cre expression in adrenal cortex, the *Prkar1a* floxed allele: *Prkar1a^loxP^ and* the knockout *Prkar1a* allele lacking exon 2: *Prkar1a^Δ2^*. Black rectangles, exons; white triangles, loxP sites; small arrows, primers used for genotyping (see Materials and Methods for details). (B) Genomic PCR experiments using primers *a, c* and *b, c* (see A) were performed to confirm all the genotypes. Corresponding phenotypes are indicated. Intact floxed allele was amplified using primers *b* and *c* and knockout allele was amplified using primers *a* and *c*. (C) Levels of the different PKA subunits were quantified by western blotting in adrenals of 10-month-old WT *vs* AdKO parous female mice. * P<0.05; ** P<0.01. (D) Top panels: *In situ* hybridization of adrenal tissue from WT (left) and AdKO (middle and with haematoxylin/eosin staining in the right) parous female mice (10-month-old) with *Prkar1a* antisense riboprobe. Lower panels: R1α protein was immunodetected in adrenal sections of WT (left) and AdKO (right) 18 month-old parous females. Arrows in AdKO indicate some of the few cortical cells in which *Prkar1a* expression is maintained. Scale bars, 50 µm.

To assess the impact of complete loss of *Prkar1a* on adreno-cortical function and initiation of PPNAD, we crossed the *Akr1b7:Cre* line [13] with mice carrying the conditional null allele *Prkar1a^loxP^* [6] to produce adrenal cortex-specific KO mice of the *Akr1b7:Cre*;*Prkar1a^loxP/loxP^* genotype (Figure 1A). In this study, these mice were referred to as AdKO mice, and wild-type mice (WT) were of the *Prkar1a^loxP/loxP^* genotype. The *Prkar1a^Δ2^* allele (KO allele) was detected by PCR in the DNA extracted from AdKO adrenals but absent from gonads and WT tissues (Figure 1B). As expected the intact conditional allele was still detected in the adrenals since Cre-mediated recombination was not supposed to occur in the whole organ but only in the cortex. Western blot and RT-QPCR analyses confirmed that *Prkar1a* gene expression was impaired in the adrenal glands of AdKO mice at both the mRNA (50% decrease) and protein levels (60% decrease) (Figure S1A and Figure 1C) when compared to WT. The 60% loss of R1α protein in adrenal tissue lysate of AdKO mice was accompanied by a significant increase in accumulation of R2β and C PKA subunits (Figure 1C), a phenomenon that is also observed in PPNAD [8]. By contrast, no significant changes were observed at the mRNA levels, indicating that upregulation of R2β and C subunits, involved a post-trancriptional mechanism (Figure S1B). We performed mRNA *in situ* hybridization and immunostaining to confirm that the decrease of *Prkar1a* gene expression was due to Cre-mediated gene ablation within the cortical compartment. As shown in Figure 1D, R1α mRNA signal was unaffected in medulla but was lost in the vast majority of cortical cells. These observations were confirmed at the protein level by R1α immunostaining (Figure 1D).

AdKO mice were born at expected Mendelian frequency and no difference in viability, weight or blood glucose values was observed up to 18 months of age when compared to WT mice (data not shown). Adrenal endocrine function and histological differentiation were explored in groups of mice of both sexes at 5, 10 and 18 months of age.

### AdKO mice progressively develop ACTH–independent Cushing's syndrome

**Figure 2:**
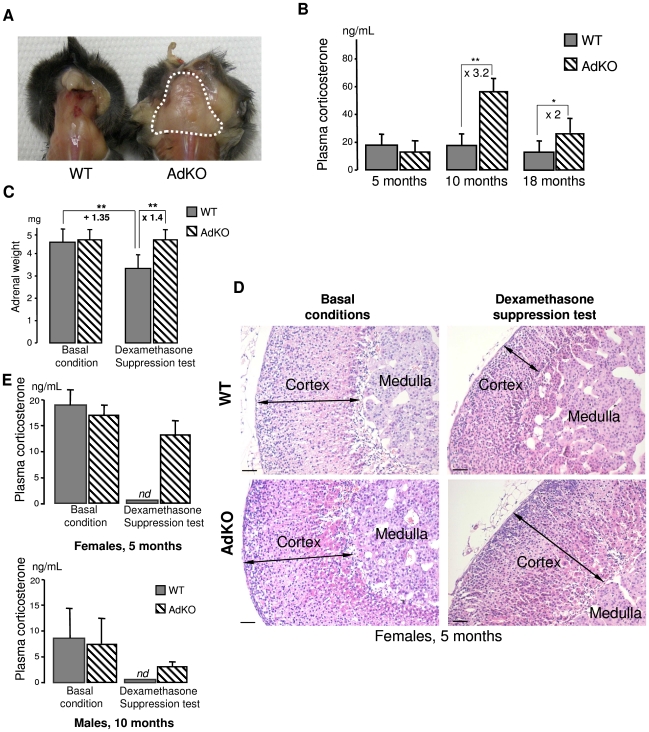
AdKO mice exhibited an age-dependent, dexamethasone-resistant increase in plasma corticosterone. (A) Abnormal accumulation of adipose tissue (dotted white line) in the back of AdKO females (10-month-old). (B) Quantitative analysis of plasma corticosterone in WT female mice compared to AdKO at 5, 10 and 18 months. (C) Mean adrenal weights of 5-month-old WT and AdKO female mice (parous) in basal conditions or after 4 days dexamethasone suppression test. (D) Representative haematoxylin and eosin adrenal staining in the same conditions as (C). Scale bars, 50 µm. (E) Quantitative analysis of plasma corticosterone in basal conditions or after dexamethasone suppression test, in 5-month-old parous females and 10-month-old males WT mice compared to AdKO mice. * P<0.05; ** P<0.01.

Visual examination of AdKO females from the age of 10 months onwards revealed neck humps formed of large accumulations of adipose tissue. This “buffalo hump” phenotype was never observed in WT females (Figure 2A) nor in males of both genotypes (not shown). Alteration of the repartition of fat depots is one of the features of “classic” Cushing's syndrome and is observed in PPNAD patients with *PRKAR1A* inactivation. In agreement with these observations, basal corticosterone levels in plasma were at least 2-fold higher in 10- and 18-month-old AdKO females than in age-matched WT, while no difference could be detected at 5 months (Figure 2B). Basal corticosterone levels were not affected in AdKO males of 5 months (8.9±6.3 ng/mL in WT *vs* 7.8±5.0 ng/mL in AdKO), 10 months (8.5±6.5 ng/mL in WT *vs* 7.8±2.1 ng/mL in AdKO) or 18 months of age (7.0±4.6 ng/mL in WT *vs* 8.2±4.2 ng/mL in AdKO). ACTH levels were measured in plasma of 10 months females. Importantly, ACTH levels measured in AdKO females were at least unchanged or tended to decrease (21±7 pg/mL in WT *vs* 14±7 pg/mL in AdKO), indicating that their basal hypercorticosteronaemia was independent of pituitary and likely resulted from primary adrenal overactivity. To explore the mechanism of hypercorticosteronaemia, AdKO mice that had not declared frank Cushing's syndrome, *i.e.* 5 months females and 10 months males, were injected with dexamethasone to induce a complete blockade of the hypothalomo-pituitary-adrenal (HPA) axis and subsequent suppression of ACTH production (Figure 2C–2E). Dexamethasone suppression test led to the expected decrease of adrenal weight (measured in females) as well as cortical atrophy in WT mice but had no effect on AdKO adrenals (Figure 2C, 2D and S2). Moreover, corticosterone levels were undetectable in plasma of WT mice after dexamethasone treatment whereas they remained unaltered in AdKO mice (Figure 2E). Finally, ACTH replacement in dexamethasone-treated mice restored corticosterone levels in WT and led to a further increase in AdKO mice, indicating that lack of R1α did not impair ACTH inducibility of steroidogenesis (Figure S3). Altogether, these data demonstrated that the adrenal glands of AdKO mice acquired the ability to secrete corticosterone in an autonomous manner leading to frank (in females) or subclinical (in males) ACTH-independent Cushing's syndrome.

### Adrenal cortex-specific ablation of *Prkar1a* increases PKA signalling

**Figure 3:**
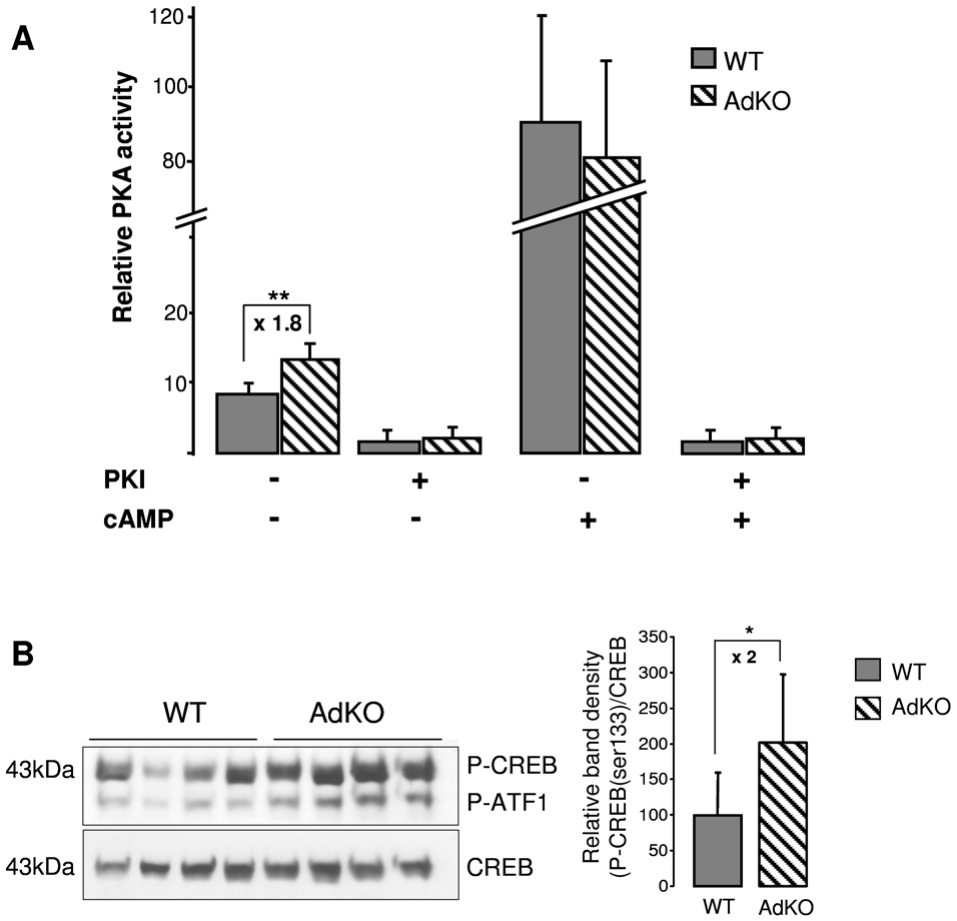
PKA activity was increased in AdKO adrenals. (A) *In vitro* quantification (arbitrary units) of the PKA catalytic activity in adrenal extracts of 10-month-old WT and AdKO females. The activity was positively or negatively regulated by cAMP, the ligand of regulatory subunits, and/or PKI, a selective inhibitor of the PKA catalytic subunits, respectively. ** P<0.01. (B) Representative CREB/P-CREB western blotting and quantification of the CREB protein phosphorylation in 10 months WT and AdKO adrenals. * P<0.05.

Glucocorticoid biosynthesis depends on the continuous ACTH stimulation of adrenal steroidogenic and detoxification genes, through the cAMP/PKA signalling pathway. We thus studied the expression level of ACTH-dependent (*Star*, *Akr1b7*, *Cyp11a1*, *Cyp11b1*) and -independent (*Cyp11b2*) genes in WT and AdKO adrenal glands (Figure S4). RT-QPCR showed that basal hypercorticosteronaemia found in 10 months AdKO females correlated with a significant increase in *Star* mRNA levels (Figure S4B). A corresponding rise of StAR protein accumulation was confirmed by western blot (Figure S4B, inset). Consistent with their milder phenotype, males did not show any significant change in the expression of steroidogenic genes. By contrast, when AdKO mice with subclinical Cushing's syndrome (5-month-old females and 10-month-old males) were submitted to dexamethasone suppression test, most of the ACTH-responsive genes remained upregulated when compared to WT (Figure S4C). As expected, *Cyp11b2* gene expression remained unchanged, showing that this response of mutant mice depended on ACTH signalling. We then checked whether *Prkar1a* ablation in AdKO mice led to the expected increase in PKA signalling, by measuring the PKA kinase activity and CREB phosphorylation on ser133 residue. Kinase assays demonstrated that basal PKA activity (in the absence of cAMP) was increased in mutant adrenals while total activity (in the presence of cAMP) remained unchanged (Figure 3A). In agreement with an increase in basal PKA activity, the amount of P-CREB in AdKO adrenals was doubled when compared to WT (Figure 3B). All these converging data demonstrated that the adrenal cortex-specific ablation of the *Prkar1a* gene led to primary pituitary-independent hypercorticosteronaemia through enhancement of PKA signalling.

### AdKO mutant mice show adrenal hyperplasia and zonation defects

**Figure 4:**
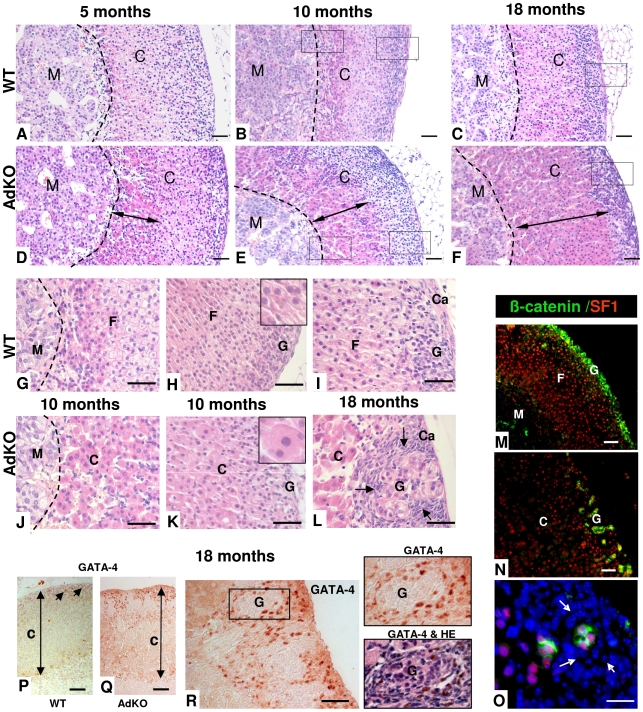
Morphological defects and progressive hyperplasia in AdKO adrenals. (A–L) Adrenals were collected from WT (A–C and G–I) and AdKO (D–F and J–L) parous females aged of 5, 10 and 18 months and stained with haematoxylin and eosin. AdKO adrenal cortex presented progressive centrifugal expansion of large eosinophilic cells, indicated by a double arrow in (D–F). In the top panels, squares delineate the higher magnifications shown in (G–I) for WT and (J–L) for AdKO adrenals. *Insets*, higher magnification illustrating the increased cell area of expanding eosinophilic cells compared to normal spongiocytes. (M–O) Adrenal sections from 18-month-old mice double-stained for β-catenin, a *zona glomerulosa* marker (in green) and for Sf1, a steroidogenic marker (in red). Nuclei were stained by Hoechst in blue. (M) WT adrenal cortex. (N) AdKO adrenal cortex. (O) Higher magnification of (N), where hyperplastic small spindle-shaped, basophilic cells lacking both staining are enlightened by arrows. (P–R) Immunodetection of the pre-tumoural marker Gata-4 in adrenal sections of parous 18-month-old females. (P) WT. (Q–R) AdKO. (R) The square delineates the higher magnifications shown in the right. Top panel: Gata-4 staining. Lower panel: same section with haematoxylin and eosin counter-staining. M, Medulla; C, Cortex; F, *zona fasciculata*; G, *zona glomerulosa*; Ca, Capsule; Scale bars, 50 µm.

Histological abnormalities consisting of large eosinophilic fœtal-like cells emerging from the innermost part of the adrenal cortex were detected in 5-month-old AdKO mice (Figure 4A and 4D). Eosinophilic cells appeared clearly hypertrophic when compared to WT spongiocytes (229±42 µm^2^ *vs* 110±15, p<0.01) (Figure 4H and 4K, insets). This hypertrophic cell population expanded centrifugally to represent more than 50% of the cortex at 10 months (Figure 4B and 4E) and most of the cortex by 18 months (Figure 4C and 4F). Simultaneously, neighbouring *zona fasciculata* cells, still arranged in tighly packed cords in 5-month-old mutant mice, became gradually disorganized in 10-month-old adrenals and appeared completely atrophic at 18 months (Figure 4G–4L). At this stage, the *zona glomerulosa* no longer appeared as a continuous layer of cells but as groups of glomeruli, isolated from each other by small hyperplastic spindle-shaped basophilic cells, arising from the subcapsular region (Figure 4L). This region contains adrenal stem/progenitor cells ensuring the continuous renewal of the adult cortex [14]–[16]. We thus assessed possible changes in the contents of adrenocortical progenitors in AdKO females of 10 and 18 months of age by quantifying expression of the progenitor-specific marker *Shh and Gli1* [16] and the potential stem cell marker *Pod1* [17]. RT-QPCR analyses showed that expression of these genes were not affected by the genotype nor the age (Figure S5), suggesting that the number of adrenocortical progenitors may not be affected in AdKO mice. The different cell populations in this area were characterized by double immunostaining for Sf1, a marker of steroidogenic lineage, and for β-catenin, a marker of subcapsular steroidogenic lineage mostly represented by *zona glomerulosa* cells [18]. Double immunostaining of WT adrenals from 18-month-old mice confirmed that both markers (Sf1 staining in the nucleus and β-catenin mostly at the cell periphery) colocalized in the cells of *zona glomerulosa* that formed a continuous layer in the outermost cortex (Figure 4M). By contrast, in age-matched AdKO adrenals, the general disorganization of the innercortex (Sf1-positive, β-catenin-negative cells) and the discontinuous aspect of the subcapsular/glomerulosa zone (Sf1/β-catenin-positive cells) were obvious (Figure 4N). A detailed view of this area showed hyperplastic spindle-shaped cells, both Sf1- and β-catenin-negative, were surrounding the Sf1- and β-catenin-positive glomeruli (Figure 4O). In previous mouse models for adrenocortical tumours, the first signs of neoplastic transformation seemed to coincide with the emergence of Gata-4 positive cells growing centripetally from the subcapsular region [15]. Thus, we examined expression of this transcription factor by immunostaining on 18-month-old adrenal sections. As shown in Figure 4P-4Q, numerous cells with Gata-4 nuclear staining could be detected within the subcapsular hyperplastic region of AdKO adrenals while, as expected, very rare positive cells were observed in WT.

**Figure 5:**
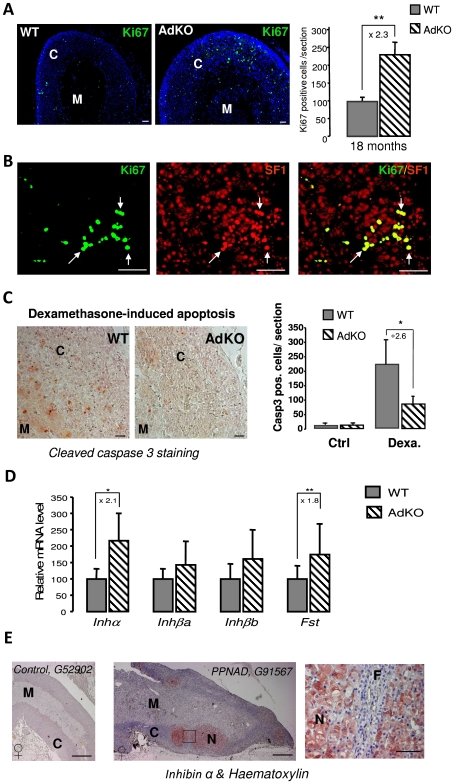
Proliferation and resistance to apoptosis in AdKO adrenals, inhibin-activin system in AdKO adrenals, and PPNAD. (A) Adrenals from 18-month-old WT and AdKO parous females were immunodetected with Ki67 proliferation marker. The corresponding statistical analysis of the number of Ki67 expressing cells in the adrenal cortex of both genotypes is shown on the right panel. ** P<0.01. (B) Co-immunodetection of Ki67 (green) and Sf1 (steroidogenic marker, red) in 18 months AdKO adrenal sections. Co-localisation of Ki67 and Sf1 stainings is shown in the right panel. (C) Representative stainings for cleaved-caspase 3 apoptosis marker in WT and AdKO adrenal sections of 5-month-old mice treated for 4 days with dexamethasone. The right panel shows the quantification of apoptotic cells (cleaved-caspase 3 positive) in adrenal cortex of untreated (Ctrl) or treated (Dexa) mice of both genotypes. * P<0.05. (D) Quantitative representation of mRNA expression (RT-QPCR) of genes encoding inhibin-activin system in 10-month-old parous female adrenals of both genotypes. Inhibin subunit (*Inhα*); Activin A and B subunits (*Inhβa* and *Inhβb*); Follistatin (*Fst*). * P<0.05; ** P<0.01. (E) Immunostaining for INHIBIN-α on human adrenal sections from control G52902 patient (female) and PPNAD G91567 patient (female) carrying germline *PRKAR1A*-inactivating mutation c.709-7del6(TTTTTA) [61]. Similar stainings were observed on two other control patients (not shown) and four other PPNAD patients (Figure S6). Square delineates the higher magnifications located on the right. N, nodule; M, medulla, F, *zona fasciculata*. Scale bars, 15 mm in entire human adrenal and 50 µm in magnification.

To determine the mechanisms leading to early hyperplasia of the large eosinophilic cells and late hyperplasia of the small spindle-shaped cells, cellular proliferation within adrenal glands of 5–18 months mice was assessed by immunodetection of Ki67 (Figure 5A). At 5 or 10 months, WT and AdKO adrenals did not show any difference in the number of Ki67 positive cells (not shown). By contrast at 18 months, the number of proliferative cells was more than doubled in mutant adrenal cortex. All Ki67-positive cells were also and thus probably corresponded to the large eosinophilic cells but not to the Sf1-negative small spindle-shaped cells (Figure 5B). The increased cell proliferation was only evident in late stage of the AdKO phenotype and then could not be sufficient to explain the hyperplasia of innercortex observed in 5-10-month-old mice. We thus tested the sensitivity of adrenocortical cells to apoptosis induced by dexamethasone injections in 5-month-old mice [19]. Although apoptotic cells, assessed by positive staining for cleaved caspase 3, were detected within the cortex of both genotypes upon dexamethasone treatment, adrenal sections from AdKO mice showed 62% less apoptotic cells than WT (Figure 5C). These data support the view that early hyperplasia observed in 5-10-month-old AdKO mice could be, at least in part, the result of decreased sensitivity to apoptosis. TGFβ superfamily members inhibin and activin play a critical role in the growth dynamics of transient zones in the developing adrenal of both human and mouse [20], [21]. The expression of genes encoding the activin subunits (*Inhβa* and *Inhβb*), the inhibin subunit (*Inhα*) and the activin-binding protein follistatin (*Fst*) were compared in WT and AdKO adrenals (Figure 5D). The mRNA levels of inhibin subunit and follistatin were 2-fold higher in AdKO adrenals whereas expression of activin subunits remained unchanged. To assess the relevance of this observation in the human adrenal, we realised an immunostaining against INHIBIN-α on sections from normal (3 patients) and PPNAD-affected adrenocortical tissues (5 patients) (Figure 5E and Figure S6). Hypertrophic cells that form the nodules were strongly stained. By contrast, no significant signal could be detected in the surrounding tissue corresponding to *zona fasciculata* nor in sections of normal adrenal tissues. Hence, in both mice and humans, R1αdepletion in adrenal cortex led to increased expression of TGFβ members known for their antagonistic effects on apoptotic action of activins [22].

### Adrenal specific ablation of *Prkar1a* leads to resurgence of foetal features

**Figure 6:**
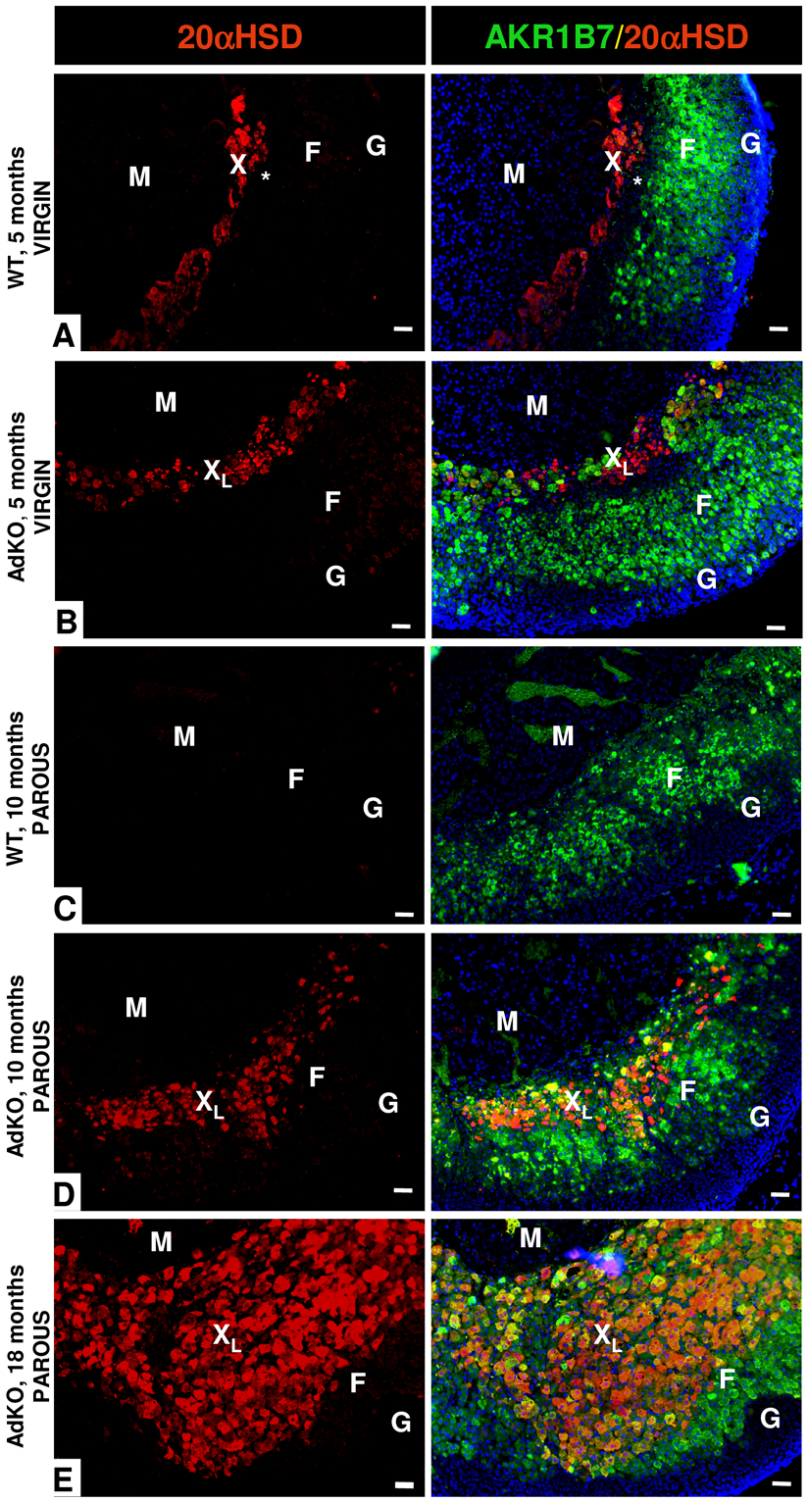
Existence of a persistent, mislocated X-like-zone in AdKO adrenals. The X-zone 20α-HSD marker (in red) and the *zona fasciculata* Akr1b7 marker (green, right column) were co-immunodetected. The two colours are merged in the right column with the Hoechst nuclei marker (blue). (A) Adrenal section of a 5-month-old nulliparous WT female. The unlabelled zone separating the X-zone (red) and the *zona fasciculata* (green) is indicated by a star (*). (B) Adrenal section of a 5-month-old nulliparous AdKO female. (C) Adrenal section of a 10-month-old parous WT female. (D) Adrenal section of a 10-month-old parous AdKO female. (E) Adrenal section of a 18-month-old parous AdKO female. M, Medulla; F, *zona fasciculata*; G, *zona glomerulosa*; X, X-zone; X_L_, X-like-zone; Scale bars, 50 µm.

Adrenocortical-specific ablation of the *Prkar1a* gene led to expansion of hypertrophic eosinophilic cells emerging from the innermost part of the cortex, adjacent to adrenal medulla (Figure 4). We hypothesized that this cell population could originate from the X-zone, a transient zone of fœtal origin that regresses during the first pregnancy in female and at puberty in male mice [23], [24]. Hence, we compared adrenal zonation in WT and mutant mice by using Akr1b7 and 20α-HSD immunostaining to delineate *zona fasciculata* and X-zone, respectively [25], [26] (Figure 6 and Figure S7). Adrenal cortex from WT virgin females showed canonical concentric organization consisting of three adjacent zones: X-zone (20α-HSD-positive and Akr1b7-negative), *zona fasciculata* (20α-HSD-negative and Akr1b7-positive) and *zona glomerulosa* (20α-HSD-negative and Akr1b7-negative) (Figure 6A). By contrast, in the adrenal gland of 5-month-old AdKO virgin females although the 20α-HSD-expressing cells remained adjacent to the medulla, the X-zone and *zona fasciculata* were now overlaping in the innermost part of the cortex, and some isolated cells co-expressed both 20α-HSD and Akr1b7 markers (Figure 6B). As expected, in 10-month-old parous WT females, X-zone had completely regressed and 20α-HSD-expressing cells were no longer detected (Figure 6C). By contrast, adrenal cortex from age-matched parous AdKO females showed a persistent large X-like-zone that has clearly expanded in a centrifugal direction (Figure 6D). At this stage, most 20α-HSD positive cells of the X-like-zone also expressed Akr1b7 and the typical packed cords organization of *zona fasciculata* was no longer observed in the Akr1b7-positive/20α-HSD-negative remaining cortex. In 18-month-old females, the X-like-zone had further expanded and now represented most of the cortex. Akr1b7-positive/20α-HSD-negative cells were repelled to adrenal periphery (Figure 6E). Interestingly, examination of the proliferative potential of the X-like-zone using double immunostaining showed that Ki67-positive/20α-HSD-positive cells could be found in both 10 and 18-month-old AdKO females (Figure S8).

Many 20α-HSD positive cells were also detected in 18-month-old AdKO males although the observed phenotype was milder than in females (Figure S9). Indeed, most 20α-HSD-expressing cells were not Akr1b7-positive and the overlap of X-like-zone with *zona fasciculata* was limited to the innermost cortex. In mouse, natural X-zone regression at puberty can be suppressed by castration of pre-pubescent males. To explore the possible reasons for the less pronounced phenotype in AdKO males, we tested whether gonadectomy occuring at 3-weeks of age (before natural X-zone regression) could accelerate the onset of the adrenal defects in 3-month-old adult males (Figure S10). 20α-HSD/Akr1b7 co-immunostaining showed that castration allowed the maintenance of a classical X-zone in WT, and of a X-like-zone overlaping fasciculata in AdKO adult males. When compared to shame-operated AdKO males of the same age, gonadectomized AdKO males showed a high number of Akr1b7-positive/20α-HSD-positive cells, these cells being never observed in gonadectomized WT males (Figure S10A). Consistently, corticosterone levels were more elevated in gonadectomized than in shame-operated AdKO males (Figure S10B). By contrast, gonadectomy had no impact on corticosterone levels in WT males. These data suggested that gonadectomy was able to amplify the phenotype in male AdKO mice.

**Figure 7:**
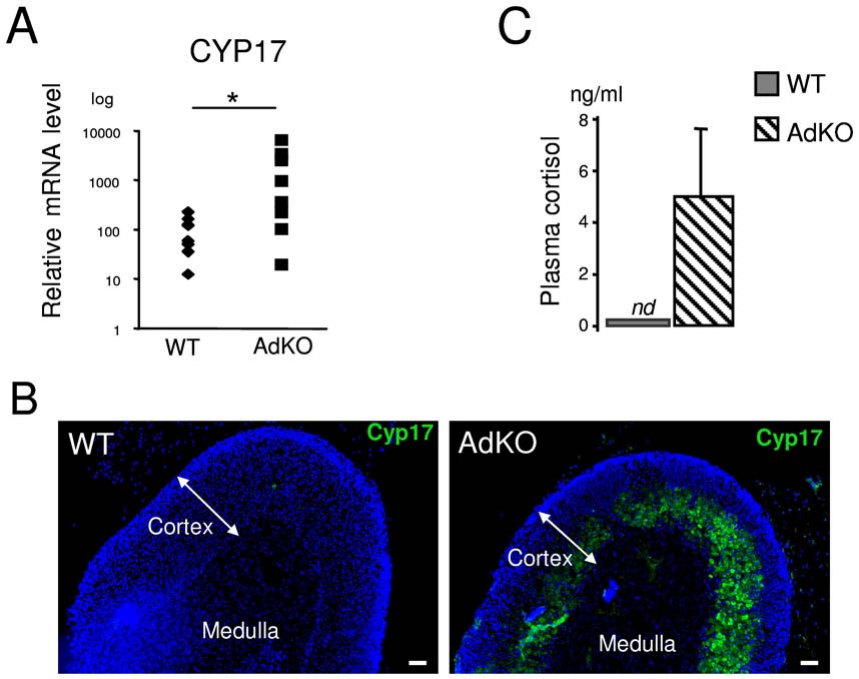
AdKO adrenals expressed Cyp17 and produced cortisol. All the experiments were realised in 10-month-old parous females. (A) Quantitative representation (RT-QPCR) of *Cyp17* mRNA levels, a foetal adrenal marker in mouse, in WT and AdKO adrenals. (B) Representative immunodetection of Cyp17 showing abnormal expression in the innercortex in AdKO compared to WT adrenals. (C) Quantitative analysis of plasma cortisol showing significant level of cortisol in AdKO compared to normal absence in WT mice. * p<0.05.

The persistence of X-zone marker suggested that foetal characteristics were maintained throughout adult life of AdKO mice. Cyp17 is a steroidogenic enzyme involved in the biosynthesis of precursors of both sex steroids and cortisol. In rodents, however, Cyp17 is only transiently expressed in the foetal adrenal and is therefore considered a foetal marker [27]. RT-QPCR analyses showed that, as opposed to WT, most adult AdKO adrenal glands expressed high levels of *Cyp17* transcripts (Figure 7A). In addition, Cyp17 positive immunostaining was detected within the innercortex of AdKO adrenal glands (Figure 7B). More importantly, this expression led to the production of a functional Cyp17 enzyme since AdKO mice produced detectable levels of cortisol (Figure 7C). Because both corticosterone and cortisol production required the continuous expression of genes encoding Cyp21 and Cyp11b1 biosynthetic enzymes, we examined whether their expressions were maintained throughout the progression of the AdKO phenotype (Figure S11). RT-QPCR analyses demonstrated that *Cyp21* and *Cyp11b1* expression levels were unchanged with age in both WT or AdKO females.

Altogether, these data demonstrate that adrenal-specific ablation of *Prkar1a* altered the differentiation program of the adult cortex by promoting the improper maintenance and centrifugal expansion of steroidogenic competent foetal-like cells, that had the capacity to proliferate and to produce glucocorticoids (cortisol and corticosterone).

## Discussion

Here, we shown that the adrenal-specific ablation of *Prkar1a*, the Carney Complex gene 1 (*CNC1*), in mouse reproduced the essential features of PPNAD observed in humans carrying *PRKAR1A* mutations. AdKO mice developed ACTH-independent Cushing's syndrome and cortical hyperplasia combined with atrophic areas that are typical hallmarks of PPNAD [1]. This mouse model definitively proves the central role of *PRKAR1A* gene defects in the etiology of PPNAD. Furthermore, the discovery of an unexpected role of *Prkar1a* in the repression of foetal features in adrenal cortex provides novel mechanistic insight into the cellular dynamics leading to definitive adrenal tissue or, when disturbed, to morbid hyperplasia.

AdKO mice phenocopied most of the features of adrenal overactivity seen in patients. From a clinical point of view, PPNAD is difficult to diagnose because Cushing's syndrome usually develops slowly. Hypercortisolism may be mild or even periodic, with no clear decrease in plasma ACTH levels [28]–[30]. Adrenal-specific disruption of *Prkar1a* triggered subclinical hypercorticosteronaemia revealed upon blockade of pituitary ACTH, in 5-month-old mice. Around one year of age, it evolved into frank Cushing's syndrome with low, but still detectable levels of plasma ACTH. Contrasting with PPNAD, we did not detect any paradoxical rise in corticosterone levels after dexamethasone injection in AdKO mice, but only a resistance to ACTH blockade. Paradoxical response would rely on increased expression of the glucocorticoid receptor (GR) that was shown to activate PKA in PPNAD nodules [31], [32]. We did not find any AdKO-dependent increase in GR expression neither by measuring mRNA levels nor by immunohistochemical analyses (not shown). This paradoxical response could likely be a feature of human cells since adrenal cultures from *Prkar1a* haploinsufficient mice did not show paradoxical dexamethasone response in perifusion experiments [32]. Another discrepancy between AdKO adrenals and PPNAD was the absence of pigmentation in the mice glands. Hyper-pigmentation in PPNAD nodules relies on the accumulation of lipofuscin and is a consequence of autophagic deficiency [33], [34]. This decreased autophagy was thought to originate from the R1α loss and consecutive activation of mTOR signalling [35]. Lipofuscin is made of aldehyde-linked protein residues that make it non-degradable and that form under chronic mild oxidative stress conditions, at a rate inversely related to the average lifespan of species [36]. We thus speculate that more efficient enzymatic defenses against reactive aldehydes forming aducts [25] and/or shorter lifespan might preserve mice from adrenal lipofuscin accumulation under R1α depletion. The cytomegalic aspect of eosinophilic cells arising from the innercortex is a hallmark of hyperplasia seen in PPNAD patients or in AdKO mice, and could be linked to unbuffered mTOR activity [35], [37] that is a prerequisite to increased cell size [38]. Works are in progress to explore the contribution of this pathway in the pathophysiology of the AdKO model.

Although they were attributed to the lack of R1α regulatory subunit of PKA, until now, the mechanisms leading to adrenal overactivity in PPNAD were not clear. The PKA heterotetrameric holoenzyme is composed of a dimer of regulatory subunits combined with two catalytic subunits. When the regulatory subunits bind cAMP, they dissociate from the catalytic subunits, which in turn, exhibit their kinase activity [39]. In previous knockout mouse models with general loss of R1α, basal PKA activity (linked to free catalytic subunits only) measured in embryos was found increased whereas total PKA activity (cAMP-stimulated) was decreased [5]. When measured in mouse embryonic fibroblasts both activities were increased [40]. The net impacts of R1α depletion on PKA activity could therefore depend on the cell type or tissue context. Consistent with these studies, specific depletion of R1α in adrenals mainly triggered a rise in basal PKA activity, attested both by increased catalytic activity and CREB phosphorylation. This resulted in a net gain of *Star* gene expression and therefore increased basal steroidogenesis. In agreement with our findings, *StAR* gene expression was found upregulated in a serial analysis of gene expression (SAGE) of PPNAD tissues [41].

The most intriguing phenotype observed during the follow-up of AdKO mice was an atypical hyperplasia of foetal-like cortex emerging at the corticomedullary junction which, over time, extended to the periphery. However, concomitant atrophy of the adult cortex resulted in net adrenal size equivalent to WT. These results were reminiscent of early histopathological studies of PPNAD, showing that micronodules seemed to arise from the medulla-cortex boundary. These were composed of eosinophilic giant cells that were surrounded by mostly atrophic cortex resulting in an otherwise normal-sized gland [1], [42], [43]. In mice, a transient cell layer, termed the X-zone, is adjacent to the medulla and regresses at puberty in males and at the first pregnancy in females [23]. Pioneer work from Morohashi‘s laboratory provided unequivocal genetic proofs that the X-zone was a remnant of foetal cortex forming before the definitive cortex and that distinct pools of precursor cells within the foetal cortex contributed to either the definitive cortex or the transient X-zone [24], [44]. One pool of precursors activated transiently the foetal adrenal-specific enhancer of *Ad4BP/Sf1* (FAdE) and contributed to the definitive cortex while the second pool maintained FAdE activated and contributed to the formation of the transient X-zone [24]. We showed previously that the developmental pattern of *Akr1b7*-Cre mediated recombination was reminiscent of that observed with the FAdE construct and that it occurred in both foetal and definitive adrenocortical cells [13]. Here, we provided evidence that loss of R1α during adrenal development resulted in two major abnormalities: unbuffered PKA activity leading to endocrine overactivity, and persistence of foetal-like cells that expanded across the adult cortex. In human, INHIBIN-α is more expressed in foetal than in adult adrenals [45]. In PPNAD, besides the hypertrophic aspect of the cells, the overexpression of INHIBIN-α specifically in the nodules could be interpreted as another sign of foetal origin of these cells. In mice, the foetal character of these hyperplastic and hypertrophic cells was attested both by persistent expression of 20α-HSD, an X-zone marker, and by re-expression of Cyp17, an enzyme otherwise restricted to the embryonic period in rodent adrenals [26], [27]. However, in contrast to natural X-zone cells that had no reported steroidogenic potential, the foetal-like hyperplastic cells of AdKO adrenals had acquired full steroidogenic competence of *zona fasciculata* cells and produced both corticosterone and cortisol. It is tempting to speculate that this (hyper)cortisolism, hitherto never described in mouse, could participate to the Cushing's syndrome of AdKO mice.

Our data demonstrated that AdKO adrenals were less sensitive to apoptosis than WT. Apoptosis mediated by TGFβ family members largely contributed to the regression of both human foetal zone and mice X-zone in which both inhibin and follistatin opposed to the apoptotic signal triggered by activins [20], [21]. This is in good agreement with our observation that inhibin-follistatin/activin transcripts ratio was augmented in AdKO adrenal glands and could therefore contribute to strengthen anti-apoptotic paracrine signals. In addition, R1α depletion could render foetal cells less sensitive to TGFβsignalling. This mechanism likely occured in human cells since we recently demonstrated that R1α knockdown in NCI-H295R adrenocortical cells enhanced their resistance to TGFβ-stimulated apoptosis [46]. Consistent with all these observations, here we showed an increased immunostaining for INHIBIN-α specifically in the nodules of PPNAD samples. Converging data in the literature highlight the importance of inhibin/activin system as a paracrine mediator of cAMP/ACTH signalling in both foetal and adult tissues [20], [47]–[49]. Accordingly, maintaining derepression of PKA activity in the adrenal glands of AdKO mice from the foetal period to adulthood would favour the maintenance of high levels of inhibin. Interestingly, in a murine cell line postulated to originate from the X-zone [50], inhibin was shown to counteract the repressive effect of activin on the *Cyp17* gene [51]. This could provide a plausible but yet-non-demonstrated mechanism for re-expression of Cyp17 in the adult mutant gland of AdKO mice.

Most adrenocortical tumours and Cushing's syndrome are more frequent in females than in males [52]. PPNAD does not escape this rule [12]. By the age of 40 years, more than 70% of the female carriers of *PRKAR1A* defects had clinical evidence of this disease, whereas only 45% of the male carriers were concerned. In addition, PPNAD was diagnosed at a younger age in females than in males. These clinical outcomes were strikingly reminiscent of the phenotype of AdKO mice that was earlier and more severe (Cushing's syndrome and hyperplasia) in females than in males. Although the influence of sex-specific hormones cannot be ruled out as suggested by the (permissive) aggravating effect of gonadectomy in AdKO males, some gender specificities of foetal cortex cells could account for these differences in mouse. First, foetal expression of *Cyp17* was nearly completely down-regulated at E14.5 in males, whereas down-regulation occurred only at birth in females [53]. Second, post-natal foetal cortex, the X-zone, regressed at puberty in males but only during first pregnancy in females. Thus, foetal cells (and among them, foetal/X-zone precursor cells) remained for a longer time in the female cortex. Since our mouse model showed that R1α loss contributed to foetal-like cortex persistence and expansion, it is reasonable to assume that enrichment in foetal cells (or foetal precursor cells) predisposes AdKO females to manifest more severe PPNAD. To our knowledge, possible gender differences in foetal cortex dynamic changes have never been addressed in human adrenals.

**Figure 8:**
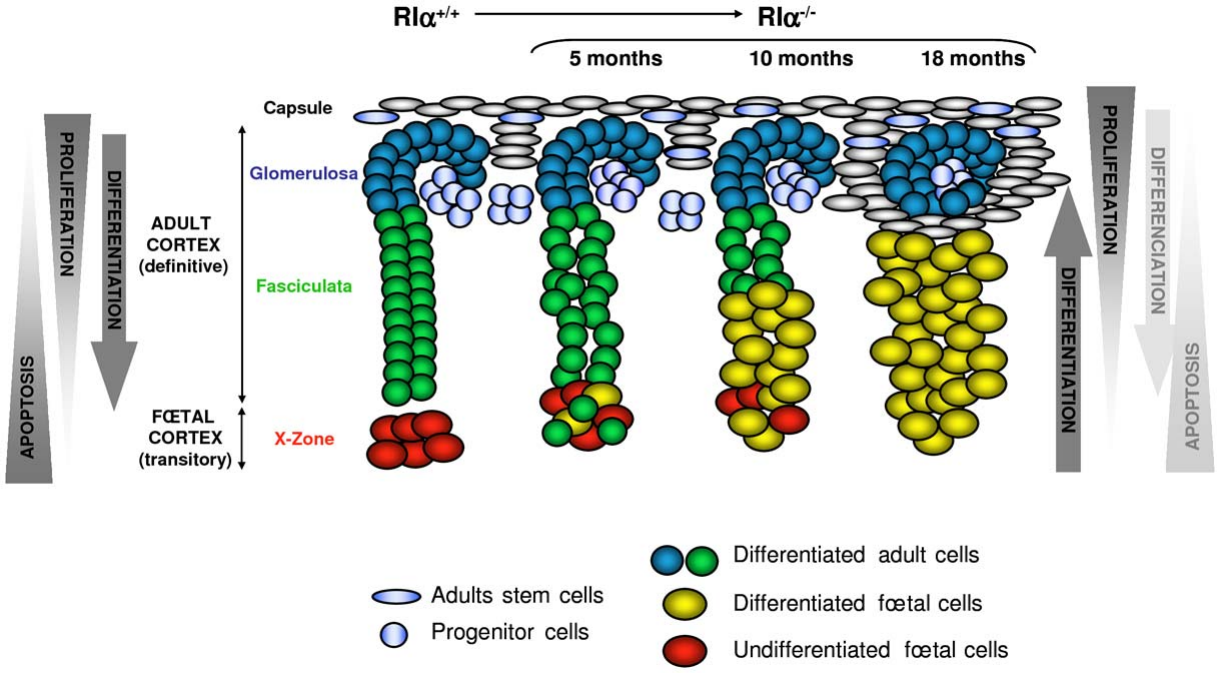
Model of dynamic changes and cell renewal in the AdKO adult cortex. *Left*, model of cell renewal and homeostatic growth maintenance of WT adrenal cortex (according to [17]). Hypothetical opposing gradients of proliferation and apoptosis leading to the set up of centripetal differentiation are schematized. *Right*, consequences of the defect of apoptosis gradient on the cellular dynamics in AdKO cortex (5-, 10-, and 18-month-old mice). Loss of R1α led to decreased apoptosis of foetal cells which were maintained, expanded across the cortex and differentiated (acquired steroidogenic competences) while definitive cortex progressively regressed.

According to the centripetal model, cell renewal in the adrenal cortex depends on a common pool of stem/progenitor cells located in the periphery (within the fibroblastic capsule and/or the *glomerulosa*) which migrate centripetally from this zone, differenciate to successively adopt all the cortical fates and finally enter into apoptosis in the innermost cortex (Figure 8). According to cell lineage-tracing studies, two populations of progenitors contributed to the formation of the adult cortex, one located in the capsule expressed *Gli1* and the second in a subcapsular position expressed *Shh* [16]. Although the role of capsule/subcapsule in the centripetal renewal of the adult definitive cortex is now fully established, there are also genetic evidences that in developing adrenal, the definitive cortex and the transient X-zone originate from different foetal precursors [24], [44].

According to the centripetal model, dividing cells are essentially present at the periphery while apoptotic cells preferentially concentrate at the inner cortex ([54], reviewed in [17]). The balance between these two opposing gradients could be essential for homeostatic maintenance of the adrenal cortex *i.e.* the establishment of a centripetal differentiation. In AdKO mice, the centrifugal expansion of foetal-like cells with proliferative potential emerging from the inner cortex and the progressive atrophy of *zona fasciculata* could indicate that this balance is perturbed. A possible model may be proposed to illustrate these observations (Figure 8). Indeed, loss of R1α allowed the maintenance of cells of foetal features, which otherwise were transient. This maintenance could result from both an improvement of their proliferative capacity and from their decreased sensitivity to apoptotis and/or alteration of the apoptotic gradient (as suggested by the increased expression of *Inhα* and *Follistatin* genes known to antagonise activins signalling). In addition, loss of R1α induced a progressive atrophy of the *zona fasciculata* in AdKO adrenals that was reminiscent to defects in cell renewal. Indeed, whereas the foetal-like cells undergo a continuous centrifugal expansion across the cortex, no gain in adrenal size was detected and no increase in cell apoptosis accompanied the concomitant atrophy of *zona fasciculata*. On the other hand, hyperplasia of non-steroidogenic subcapsular spindle-shaped cells was observed in elder mice (18 months) and their accumulation eventually affected the integrity of *zona glomerulosa*. At least two non-exclusive mechanisms could account for defective cell renewal in the definitive cortex of AdKO mice: depletion of progenitor cells or impaired capacity of these progenitors to undergo centripetal differentiation and clonal replenishment of the cortex. The latter mechanism seems more likely in our model. Indeed, the expression levels of markers for stem/progenitor cells (Figure S5) were unaltered in AdKO mice suggesting that progressive atrophy of the definitive cortex was not due to their depletion. Similar to observations made in most mouse adrenal tumour models, with age, adrenal glands of AdKO mice accumulated subcapsular Gata-4-positive spindle-shaped cells that are supposed to descend from multipotent progenitors capable to engage toward adrenal or gonadal fates [15], [55], [56]. In AdKO mice, late accumulation of Gata-4-positive cells could reflect an incapacity for the progenitor cells to properly differenciate into adrenocortical cells. This would prevent efficient renewal of the definitive cortex, which as a result, would become atrophic over time. An other hypothesis emerges from a recent report of Hammer and colleagues showing that inhibin-α prevented aberrant proliferation and differentiation of subcapsular adrenocortical progenitor cells [57]. Indeed, as opposed to *Inhα^−/−^* mice, adrenal glands of AdKO mice have increased inhibin-α expression. This is consistent with PPNAD samples and could therefore decrease proliferation/differentiation of progenitors. The possible dual role for inhibin-α in enhancing survival of foetal cells and impeding renewal of definitive cortex will have to be demonstrated in AdKO mice in an *Inhα^−/−^* context. In a symmetric point of view, the slow but continuous centrifugal expansion of hypertrophic foetal-like cells, would imply that a reservoir of foetal cortex precursor cells could lay in the juxtamedullary region and that differentiating steroidogenic foetal cells could emerge and replenish the cortex (Figure 8). Lineage tracing experiments will be required to confirm this hypothesis.

By developing a mouse model of PPNAD, we established for the first time that *Prkar1a*, the Carney Complex gene 1, not only controls adrenocortical endocrine activity but also prevents the maintenance of foetal remnants. The loss of R1α acts, at least, by increasing PKA activity and possibly by PKA independent effects mediated through alteration of protein interactions that remain to be deciphered [35], [58]. The data existing in the literature and our present results strongly suggest a role for the inhibin-activin signalling pathway in the progression of the disease. Adrenal hyperplasia observed in PPNAD is classified as a neoplastic lesion. Although we showed that R1α loss induced tumoural conditions in adrenal glands (resistance to apoptosis, cell hypertrophy, mild proliferation), profound alterations in zonal differentiation and cell renewal suggest that PPNAD should also be considered as a developmental disease.

## Materials and Methods

### Human PPNAD tissue sections

Informed signed consent for the analysis of adrenal tissue and for genetic diagnosis was obtained from the patients and the study was approved by an institutional review board (Comité Consultatif de Protection des Personnes dans la Recherche Biomédicale, Cochin Hospital, Paris). PPNAD paraffin sections were performed from adrenal samples of patients with isolated PPNAD or PPNAD with Carney complex who underwent bilateral adrenalectomy for ACTH-independent Cushing's syndrome. All patients carried germline inactivating mutations of the *PRKAR1A* gene.

### Animals and hormonal treatments

Animal studies were done in agreement with standards described by the NIH Guide for Care and Use of Laboratory Animals as well as with the local laws and regulations applicable to animal manipulations in France. For all analyses, groups of 17–20 mice of each genotype (WT and AdKO) and each age (5, 10 and 18 months) were constituted. Adult mice were injected *s.c.* with vehicle (sesame oil), dexamethasone acetate for 4 days (75 µg twice daily; Sigma-Aldrich, L'Isle d'Abeau Chesnes, France) and injected *i.m.* with long-acting ACTH (1.2 U, Synacthene, Novartis Pharma S.A., Rueil-Malmaison, France) the day before and in the morning of the experiment.

### Mouse genotyping

Mouse genomic DNA (from tail, adrenal or gonad) was extracted and analyzed by PCR. Genotyping for the 0.5 *Akr1b7*-Cre transgene was carried out using the following conditions: 94°C, 45 s; 55°C, 45 s; 72°C, 45 s for a total of 40 cycles (primers: 5′-CCTGGAAAATGCTTCTGTCCG-3′; 5′-CAGGGTGTTATAAGCAATCCC-3′) and *Prkar1a^loxP/loxP^* intact or knockout allele were genotyped using the following conditions: 94°C, 90 s; 58°C, 90 s; 72°C, 90 s for a total of 35 cycles (primer *a*: 5′- CACTGCAGGGGCCTATTTTA -3′; primer *b*: 5′-TGTCTAGCTTGGGGTGGACT-3′, primer *c*: 5′-CATCCATCTCCTATCCCCTTT-3′).

### Analysis of hormone levels

Mice were sacrified by decapitation at 8–9 am with minimum handling (within 1 min), trunk blood was collected in eppendorf tubes containing 5 µL EDTA 0.5 M and placed immediately at 4°C. Samples were spun down at 4000 g for 5 min at 4°C and the resultant plasma was stored at −20°C for corticosterone or cortisol analysis, or at −80°C for ACTH analysis.

Corticosterone concentrations in plasma were determined by radioimmunoassay (RIA) using a commercially available kit (ICN Biomedicals, Orsay, France). ACTH dosage in plasma were performed by solid-phase, two-site sequentiel chemiluminescent immunometric assay (Siemens Healthcare Diagnostic SAS, Saint-Denis, France) using an Immulite 2000 analyzer. Cortisol concentrations were determined by electrochemiluminescence immunoassay (Roche Diagnostic, Meylan, France) using a Modular Analytics E170 analyzer.

### PKA activity

PKA activity was quantified in freshly dissected adrenals using the following commercial kit: PepTag assay for non-radioactive detection of cAMP-dependant protein kinase (Promega Corp., Charbonnière, France).

### Quantitative reverse-transcription polymerase chain reaction

Total RNA and DNA (for genotype confirmation) were isolated from tissue with the Qiagen DNA/RNA Mini kit (Qiagen, Courtaboeuf, France). Total RNAs (1 µg) were reverse-transcribed by Moloney murine leukaemia virus reverse transcriptase (Promega Corp., Charbonnière, France) according to the manufacturer's instructions. Quantitative real-time PCR was performed using the iCycler BioRad system and BioRad IQ5 optical system software (BioRad, Marnes-la-Coquette, France) under standard conditions (40 cycles of 95°C for 15 seconds and 60°C for 60 seconds). All primer/probe sets were obtained from Applied Biosystems: *Prkar1a, Prkar1b, Prkar2a, Prkar2b, Prkaca, Star, Akr1b7, Cyp11a1, Cyp11b1, Cyp11b2, Cyp21, Shh, Gli-1, Pod-1, Ppib, Cyp17, Inhα, Inhβa, Inhβb, Fst* (Applied Biosystems, Courtaboeuf, France). For quantification of transcripts, all PCR were performed in triplicate and the DCt method was used to calculate mRNA levels relative to a *Peptidylprolyl isomerase B (Ppib)* standard.

### *In situ* hybridization

A 442 bp 3′untranslated part of the *Prkar1a* cDNAs was amplified using the following primers: 5′-GGGCGTTGGAATTACTGAGA-3′; 5′-CTCCCAAATAGAACCCGACA -3′; and subcloned in pGEM-T easy vector (Promega Corp., Charbonnière, France). Antisense riboprobes were synthesized and labelled with digoxigenin (Boehringer Mannheim, Mannheim, Germany). Adrenals were fixed in 4% paraformaldehyde overnight, embedded in paraffin and sectioned. Sections were treated for 15 min with proteinase K (3 µg/ml) at room temperature and washed with glycine (2 mg/ml) and then with PBS. They were fixed with 4% paraformaldehyde for 5 min and washed with PBS. Samples were incubated in hybridization mix (50% formamide; 4x SSC; 10% Dextran sulphate; 1x Denhart's; Salmon sperm DNA 250 µg/ml; tRNA 250 µg/ml) for 1 h at 42°C. Digoxygenin labelled probe was added to the hybridization mix and incubated overnight at 42°C. Slides were then treated to a series of washes in 2x SSC and 1x SSC at 42°C and 0.2x SSC at room temperature. Sections were washed in buffer 1 (150 mM NaCl; 100 mM Tris, pH 7.5), blocked by Boehringer blocking reagent in buffer 1 then incubated 1 h at room temperature with peroxidase-conjugated anti-digoxygenin antibody. After several washes in buffer 2 (150 mM NaCl; 100 mM Tris, pH 9.5; 5 mM MgCl_2_), peroxidase activity was detected by incubation with 0.18 mg/ml BCIP and 0.34 mg/ml NBT in buffer 2. *In situ* hybridization slides were observed and photographed on an Axiophot microscope (Carl Zeiss, Zurich, Switzerland).

### Histology and immunostaining

Adrenals were fixed overnight in 4% PFA and embedded in paraffin. Sections were then cut and deparaffinized in Histoclear. For general morphology, sections were stained with haematoxylin and eosin.

For mouse-anti-human-INHIBIN-α immunodectection, unmasking solution was sodium citrate buffer 10 mM pH 6, Tween 0.05%. For co-localisation experiments of Akr1b7/Ki67 with 20α-HSD, the following protocol of limit detection was used: deparaffinised sections were incubated for 20 min at 95°C with Unmasking Solution (Vector Laboratories, Peterborough England). For the first detection, rabbit-anti-Akr1b7 antibody [59] (1/1000) or rabbit-anti-Ki67 (1/500, Thermo Fischer Scientific, Elancourt, France) was revealed using a secondary biotinylated goat anti-rabbit antibody, Vectastain ABC amplification kit (Vector Laboratories, Peterborough England) and TSA fluorescein HRP substrate (Perkin Elmer, Courtaboeuf, France). For the second detection, slides were incubated with a rabbit-anti-20α-HSD antibody at 1/2000 (kind gift from Dr Y. Weinstein, Ben-Gurion University, Israël) revealed by goat anti-rabbit Alexa 555 at 1/1000 (Molecular probes, Cergy Pointoise, France). Sections were then incubated 5 min with Hoechst at 1 µg/ml (Sigma-Aldrich, L'Isle d'Abeau Chesnes, France), rinsed, mounted in PBS-glycerol, and photographed on an Axiophot microscope (Carl Zeiss, Zurich, Switzerland).

The following antibodies: Mouse-anti-R1α (1/50, BD Biosciences, Le pont de Claix, France); rabbit-anti-Sf1 (1/1000, kind gift from Dr K. Morohashi, Kyushu University, Japan), rabbit-anti-Cyp17 (1/5000, kind gift from Dr A. Conley, University of California, USA); rabbit-anti-cleaved-Caspase-3 (1/400, Cell signalling, Saint-Quentin-en-Yvelines, France); goat-anti-GATA-4 (1/100, Tebu-Santa Cruz, Le Perray en Yvelines, France), mouse-anti-β-catenin (1/500, BD Biosciences, Le pont de Claix, France), mouse-anti-human-INHIBIN-α (1/75, AbD Serotec, Oxford, UK) were detected using the same protocol as Akr1b7. The secondary biotinylated antibodies were donkey anti-goat to detect Gata-4 and sheep anti-mouse to detect R1α and β-catenin. Gata-4 detection was performed using the Novared Kit (Abcys, Paris, France).

For the double staining β-catenin/Sf1, 20 min in 0.02% HCl are necessary to abolish the rest of the peroxidase activity after the first immuno-reaction. Detection of Cyp17 was done without incubation in unmasking solution. An InSitu Pro VSi (Intavis AG) automated processor was used for immunodetection.

### Western blot analysis

Adrenal samples and western blotting were done as described previously [60]. The Primary antibodies were used at the following dilutions: rabbit-anti-StAR (1/5000, kind gift from Dr Stocco, Texas Tech University Health Sciences Center, USA); mouse-anti-R1α (1/500); mouse-anti-R2α (1/1000); mouse-anti-R2β (1/1000); mouse-anti-Cαβ (1/1000, BD Biosciences, Le Pont de Claix, France), rabbit-anti-CREB (1/1000), rabbit-anti-P-CREB (1∶1000, Cell signalling, Saint-Quentin-en-Yvelines, France); rabbit-anti-βTubulin (1/1000, Sigma-Aldrich, L'Isle d'Abeau Chesnes, France). Quantification of western blot signals was performed using the Quantity One software (Biorad, Marnes la Coquette, France).

### Statistical analysis

For statistical analysis, a Student *t* test was performed to determine whether there were differences between the two groups. Mann-Whitney test was used in Figure 7A. A *P* value of 0.05 was considered significant.

## Supporting Information

Figure S1 Quantification of mRNA levels of the PKA subunits in 10-month-old, WT and AdKO mice adrenals. A, Quantitative (RT-QPCR) representation of R1α subunit mRNA expression in female (parous) and male adrenals of both genotypes. A significant decrease was detected in AdKO when compared to WT, as expected, ** p<0.01. Insets show levels of R1α subunit analysed by western blotting in adrenals. B, Quantitative (RT-QPCR) representation of mRNA expression of the different PKA subunits in female adrenals of both genotypes.
(0.18 MB TIF)

Figure S2 Morphological defects and dexamethasone-resistance in AdKO adrenals of male mice. Representative haematoxylin and eosin adrenal staining of 10 and 18-month-old males of WT and AdKO genotype, in basal conditions or after 4 days dexamethasone suppression test. Insets, higher magnification illustrating the increased cell size of expanding eosinophilic cells compared to normal spongiocytes. The dotted line delineates the cortex-medulla boundary. Double arrows indicate the cortex. Scale bars, 50 µm.
(5.01 MB TIF)

Figure S3 Sensitivity to ACTH of plasma corticosterone levels in WT and AdKO adrenals. Quantitative analysis of plasma corticosterone in dexamethasone-treated mice (5-month-old parous females, 5- and 10-month-old males) with or without ACTH replacement. * p<0.05. NS: statistically non significant.
(0.19 MB TIF)

Figure S4 ACTH responsive genes were maintained up-regulated in AdKO adrenals. A, efficiency of the dexamethasone suppression test on the expression of genes implicated in steroidogenesis or detoxification in 5-month-old WT females (parous) and 10-month-old WT males. B–C, Quantitative representation of mRNA levels of genes involved in steroidogenesis or detoxification: *Star, Akr1b7, Cyp11a1, Cyp11b1, Cyp11b2.* RT-QPCRs were done with adrenal mRNA from WT and AdKO 10-month-old parous females and males in basal conditions (B) and from WT and AdKO 5-month-old parous females and 10-month-old males treated with dexamethasone (C). *, P<0.05; ** P<0.01. Inset, western blot showing basal up-regulation of StAR protein in AdKO adrenals from 10-month-old females.
(0.40 MB TIF)

Figure S5 Maintenance of progenitor cell markers in AdKO adrenals. Quantitative representation of mRNA levels of the genes: *Shh, Gli-1 and Pod-1*. RT-QPCRs were done using adrenal mRNA from WT and AdKO mice of 10 and 18-month-old females (parous).
(0.11 MB TIF)

Figure S6 *INHIBIN-α* was overexpressed in the adrenal nodules of PPNAD patients. INHIBIN-α was immunodetected (in brown) in adrenal sections of two males (top panels) and two females (lower panels) PPNAD patients and counter-stained with haematoxylin (blue). Scale bars, 50 µm.
(4.44 MB TIF)

Figure S7 Existence of a persistent X-like-zone in AdKO female adrenals. The X-zone 20α-HSD marker (in red) was immunodetected, and merged in the right column with the Hoechst nuclei marker (blue, right column). A–B, Adrenal sections of a 5-month-old parous WT and AdKO female. The arrows indicate cells expressing 20α-HSD. C–D, Adrenal sections of a 10-month-old virgin WT and AdKO female. E–D, Adrenal sections of a 18-month-old virgin WT and AdKO female. C, cortex; M, Medulla; X, X-zone; X_L_, X-like-zone; Scale bars, 50 µm.
(3.95 MB TIF)

Figure S8 Evidence for cell proliferation in the X-like-zone of AdKO mice adrenals. In 10 and 18-month-old parous AdKO females, the X-zone 20α-HSD marker (in red) and the Ki67 proliferation marker (in green) were co-immunodetected and merged with the Hoechst nuclei marker (blue). Double-stained cells are outlined by arrows. X_L_, X-like-zone; Scale bars, 20 µm.
(1.13 MB TIF)

Figure S9 Existence of a persistent, mislocated X-like-zone in male AdKO adrenals. The X-zone 20α-HSD marker (in red) and the *zona fasciculata* Akr1b7 marker (green, right column) were immunodetected. The two colours are merged in the right column with the Hoechst nuclei marker (blue). A, Adrenal section of a 18-month-old WT male. No staining for 20α-HSD was shown. B, Adrenal section of an 18 month-old AdKO male. Cells doubled-stained for Akr1b7 and 20α-HSD were detected, as in AdKO females, indicating the presence of a pathological X-like-zone in AdKO male adrenals. M, Medulla; F, *zona fasciculata*; G, *zona glomerulosa*; X, X-zone; X_L_, X-like-zone; Scale bars, 20 µm.
(1.37 MB TIF)

Figure S10 Castration in AdKO males increased the size of persistent, mislocated X-like-zone and plasma corticosterone levels. WT and AdKO males were castrated at 3 weeks and kept for sacrifice at 3 months of age. A, Co-immunostaining for Akr1b7 (green) and 20α-HSD (red) were realised on adrenal sections of control (left column) and castrated mice of WT and AdKO genotypes. B, Quantitative analysis of plasma corticosterone in control and castrated 3-month-old males of WT and AdKO genotypes. M, Medulla; C, cortex; X, X-zone; X_L_, X-like-zone; Scale bars, 20 µm. *, P<0.05.
(1.91 MB TIF)

Figure S11 Maintenance of steroidogenesis markers in aging AdKO adrenals. Quantitative representation of mRNA levels of the genes: *Prkar1a* (control), *Cyp21*, *Cyp11b1*. RT-QPCRs were done with adrenal mRNA from WT and AdKO mice in 10 and 18-month-old females (parous). *, P<0.05. NS: statistically non significant.
(0.09 MB TIF)
