# Supplementary figures and images for: Cushing's Syndrome and Fetal Features Resurgence in Adrenal Cortex–Specific Prkar1a Knockout Mice
Source: PLoS Genet. 2010 Jun 10;6(6):e1000980. doi: 10.1371/journal.pgen.1000980 (PMC2883593; doi:10.1371/journal.pgen.1000980)

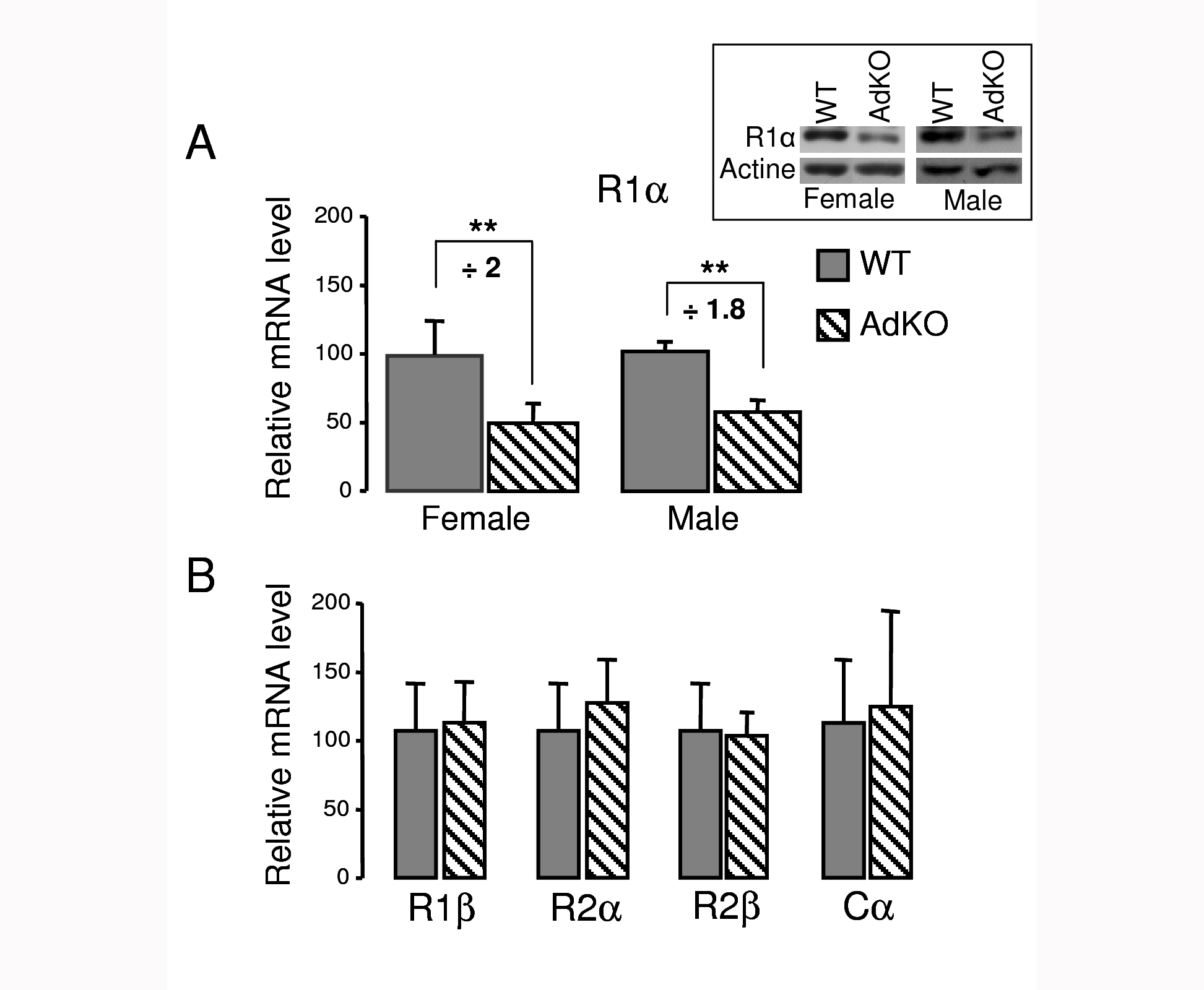

Supplement: Figure S1 — Quantification of mRNA levels of the PKA subunits in 10-month-old, WT and AdKO mice adrenals. A, Quantitative (RT-QPCR) representation of R1α subunit mRNA expression in female (parous) and male adrenals of both genotypes. A significant decrease was detected in AdKO when compared to WT, as expected, ** p<0.01. Insets show levels of R1α subunit analysed by western blotting in adrenals. B, Quantitative (RT-QPCR) representation of mRNA expression of the different PKA subunits in female adrenals of both genotypes. (0.18 MB TIF) [file pgen.1000980.s001.tif]

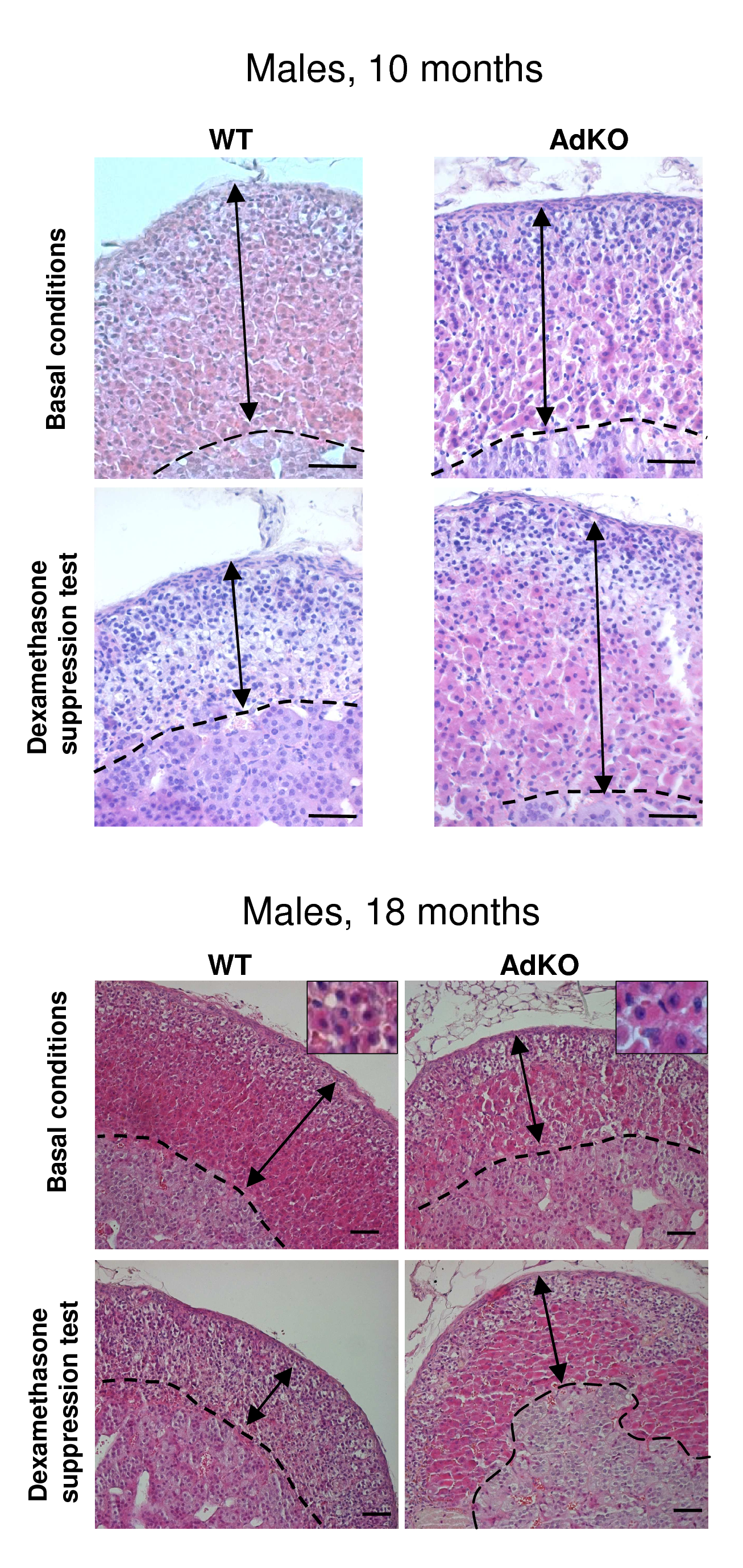

Supplement: Figure S2 — Morphological defects and dexamethasone-resistance in AdKO adrenals of male mice. Representative haematoxylin and eosin adrenal staining of 10 and 18-month-old males of WT and AdKO genotype, in basal conditions or after 4 days dexamethasone suppression test. Insets, higher magnification illustrating the increased cell size of expanding eosinophilic cells compared to normal spongiocytes. The dotted line delineates the cortex-medulla boundary. Double arrows indicate the cortex. Scale bars, 50 µm. (5.01 MB TIF) [file pgen.1000980.s002.tif]

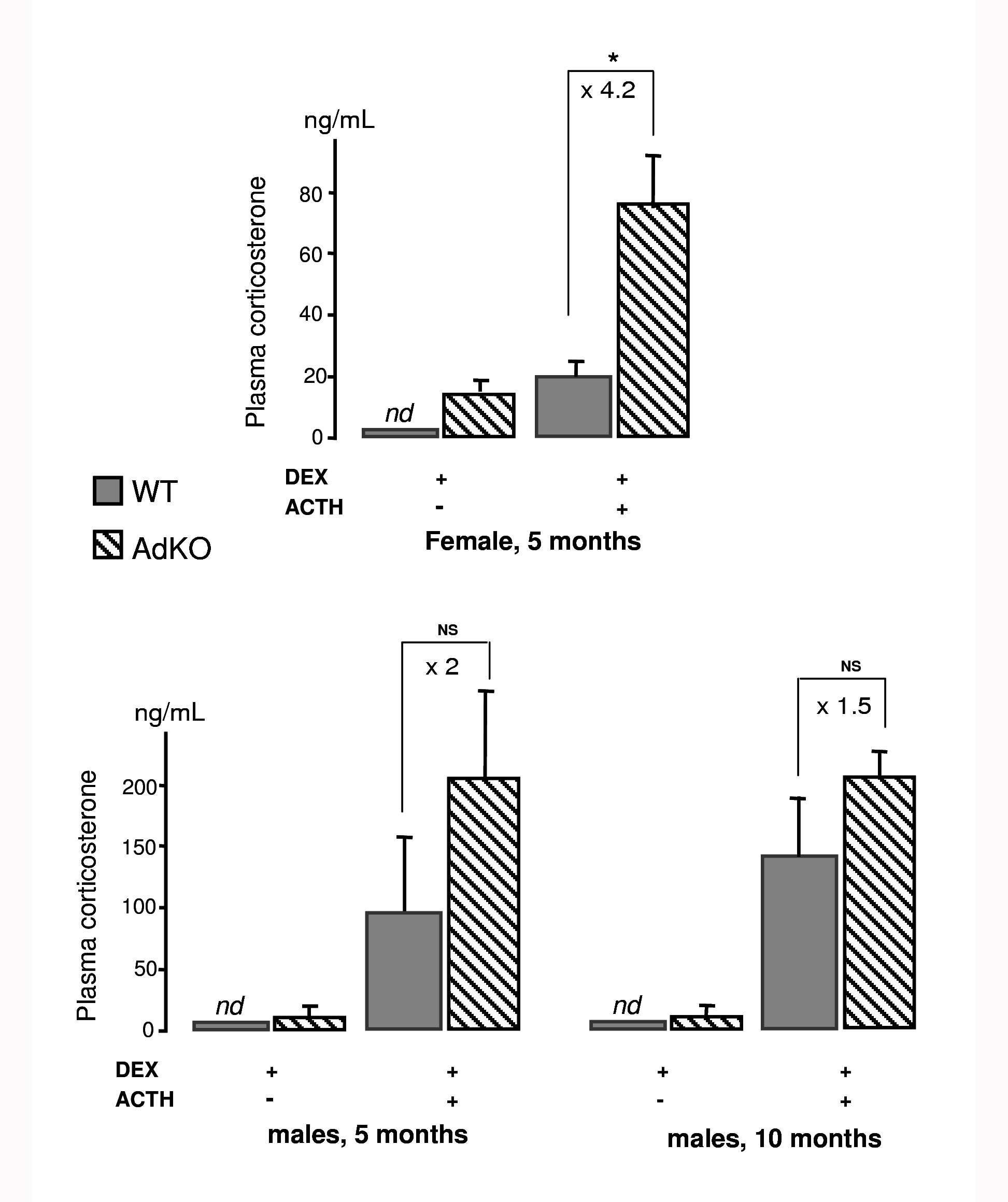

Supplement: Figure S3 — Sensitivity to ACTH of plasma corticosterone levels in WT and AdKO adrenals. Quantitative analysis of plasma corticosterone in dexamethasone-treated mice (5-month-old parous females, 5- and 10-month-old males) with or without ACTH replacement. * p<0.05. NS: statistically non significant. (0.19 MB TIF) [file pgen.1000980.s003.tif]

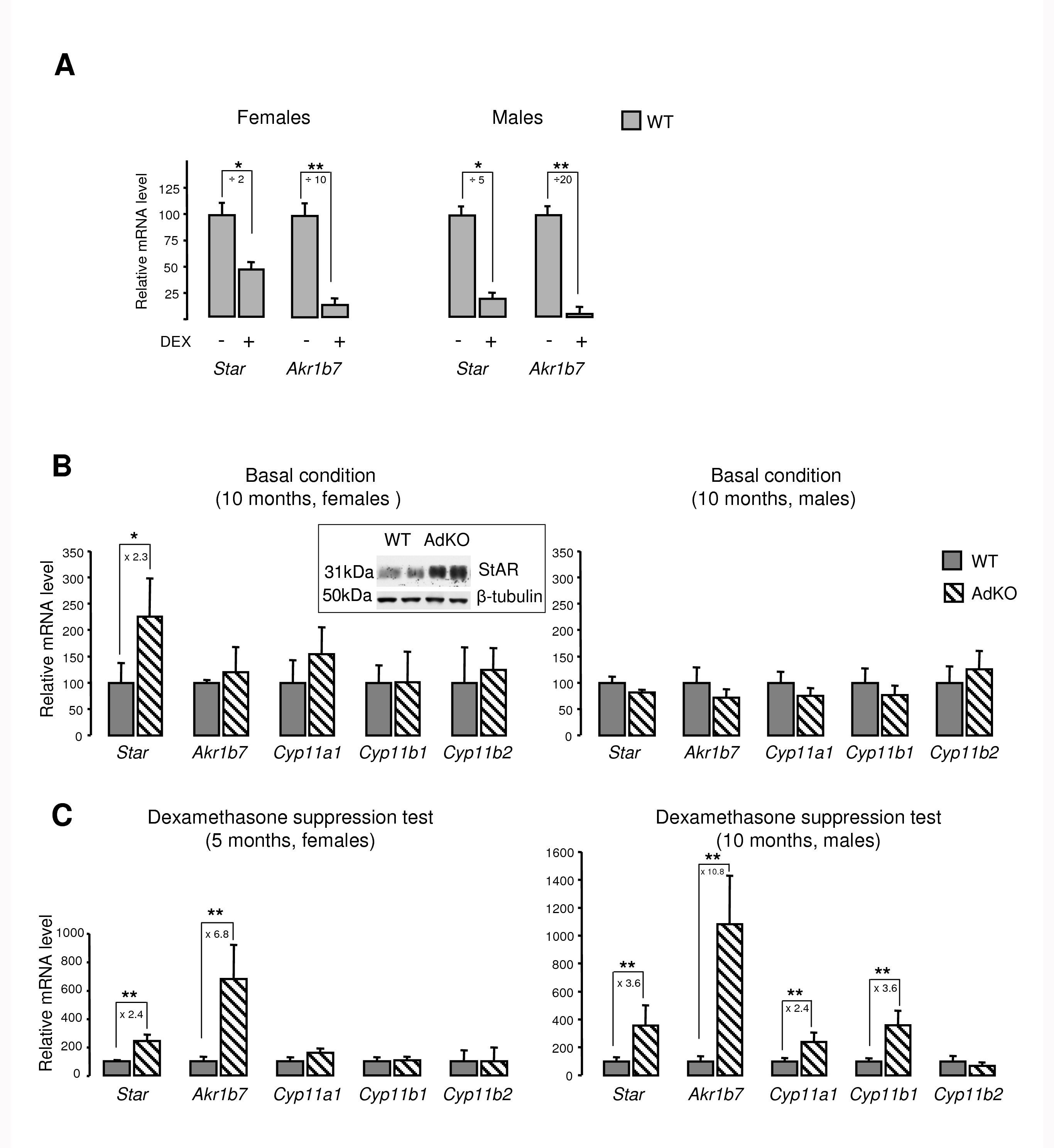

Supplement: Figure S4 — ACTH responsive genes were maintained up-regulated in AdKO adrenals. A, efficiency of the dexamethasone suppression test on the expression of genes implicated in steroidogenesis or detoxification in 5-month-old WT females (parous) and 10-month-old WT males. B–C, Quantitative representation of mRNA levels of genes involved in steroidogenesis or detoxification: Star, Akr1b7, Cyp11a1, Cyp11b1, Cyp11b2. RT-QPCRs were done with adrenal mRNA from WT and AdKO 10-month-old parous females and males in basal conditions (B) and from WT and AdKO 5-month-old parous females and 10-month-old males treated with dexamethasone (C). *, P<0.05; ** P<0.01. Inset, western blot showing basal up-regulation of StAR protein in AdKO adrenals from 10-month-old females. (0.40 MB TIF) [file pgen.1000980.s004.tif]

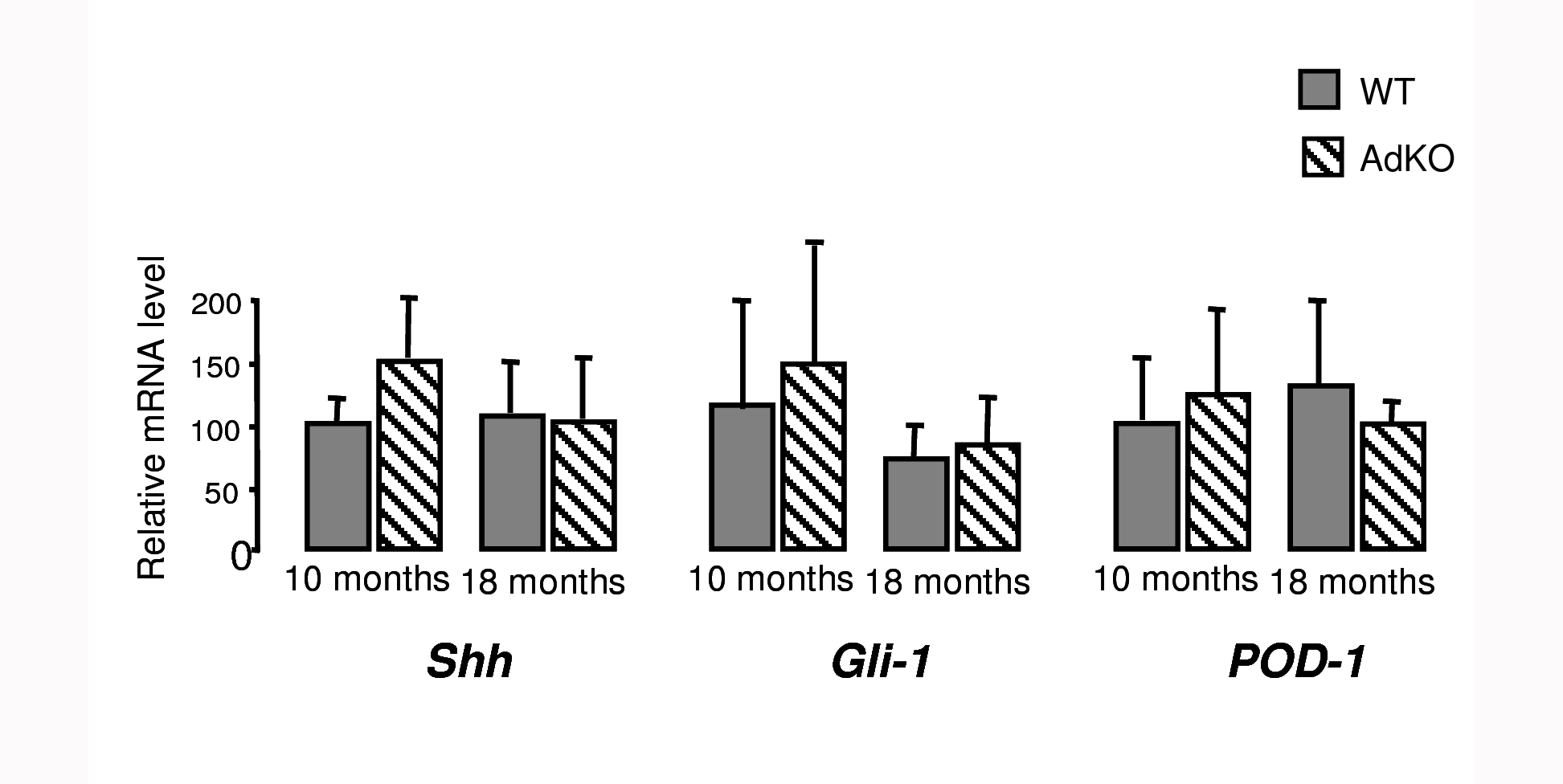

Supplement: Figure S5 — Maintenance of progenitor cell markers in AdKO adrenals. Quantitative representation of mRNA levels of the genes: Shh, Gli-1 and Pod-1. RT-QPCRs were done using adrenal mRNA from WT and AdKO mice of 10 and 18-month-old females (parous). (0.11 MB TIF) [file pgen.1000980.s005.tif]

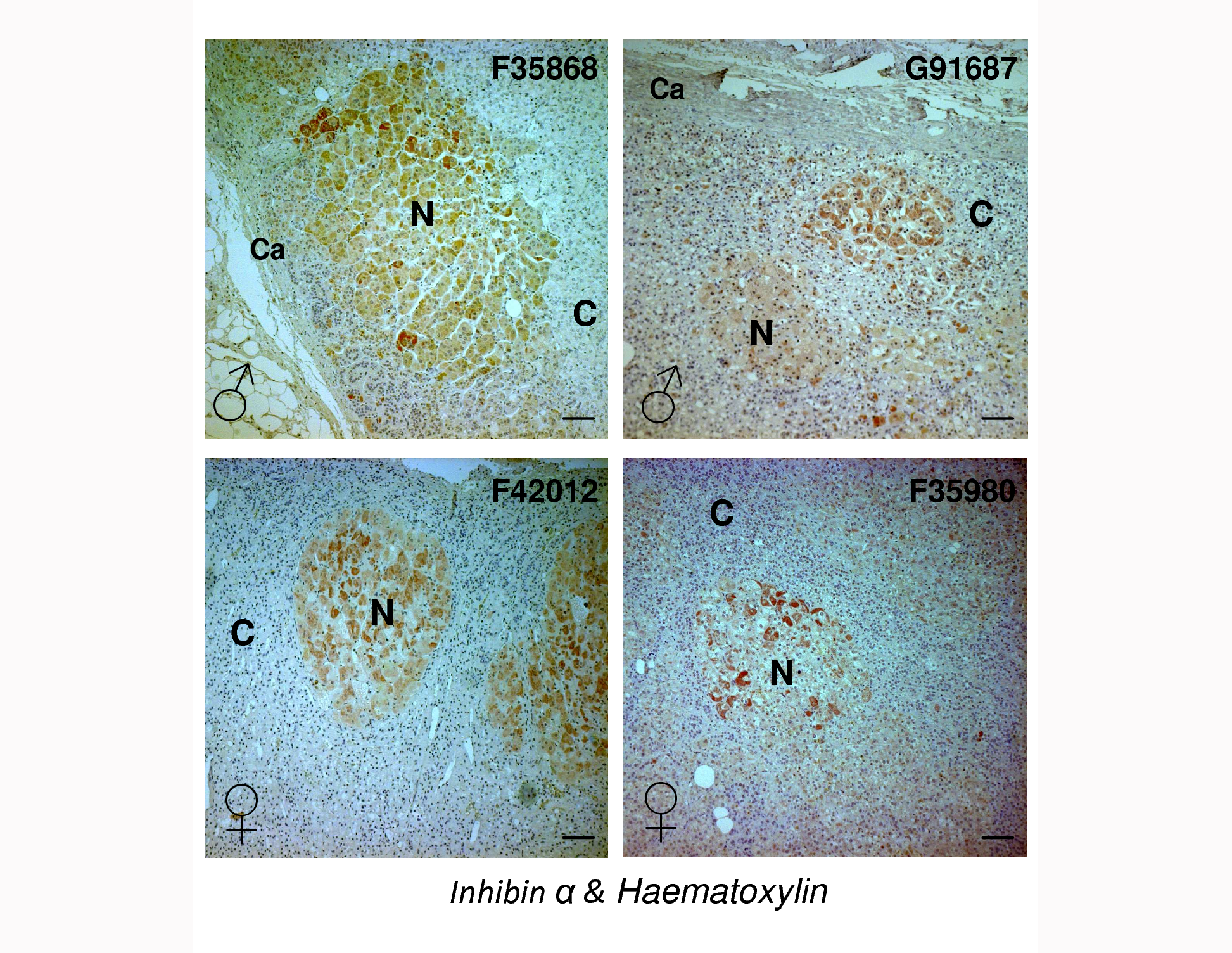

Supplement: Figure S6 — INHIBIN-α was overexpressed in the adrenal nodules of PPNAD patients. INHIBIN-α was immunodetected (in brown) in adrenal sections of two males (top panels) and two females (lower panels) PPNAD patients and counter-stained with haematoxylin (blue). Scale bars, 50 µm. (4.44 MB TIF) [file pgen.1000980.s006.tif]

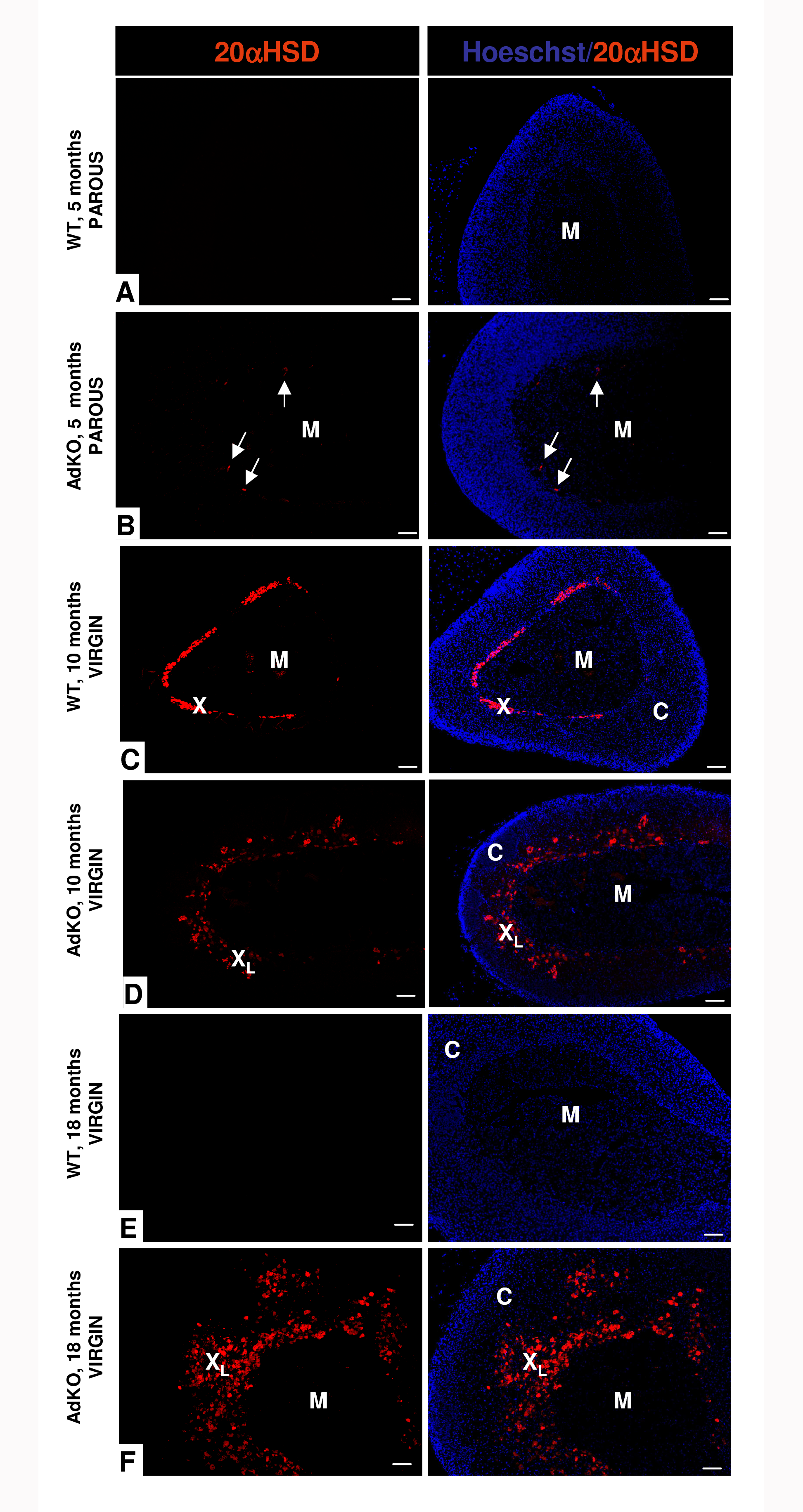

Supplement: Figure S7 — Existence of a persistent X-like-zone in AdKO female adrenals. The X-zone 20α-HSD marker (in red) was immunodetected, and merged in the right column with the Hoechst nuclei marker (blue, right column). A–B, Adrenal sections of a 5-month-old parous WT and AdKO female. The arrows indicate cells expressing 20α-HSD. C–D, Adrenal sections of a 10-month-old virgin WT and AdKO female. E–D, Adrenal sections of a 18-month-old virgin WT and AdKO female. C, cortex; M, Medulla; X, X-zone; XL, X-like-zone; Scale bars, 50 µm. (3.95 MB TIF) [file pgen.1000980.s007.tif]

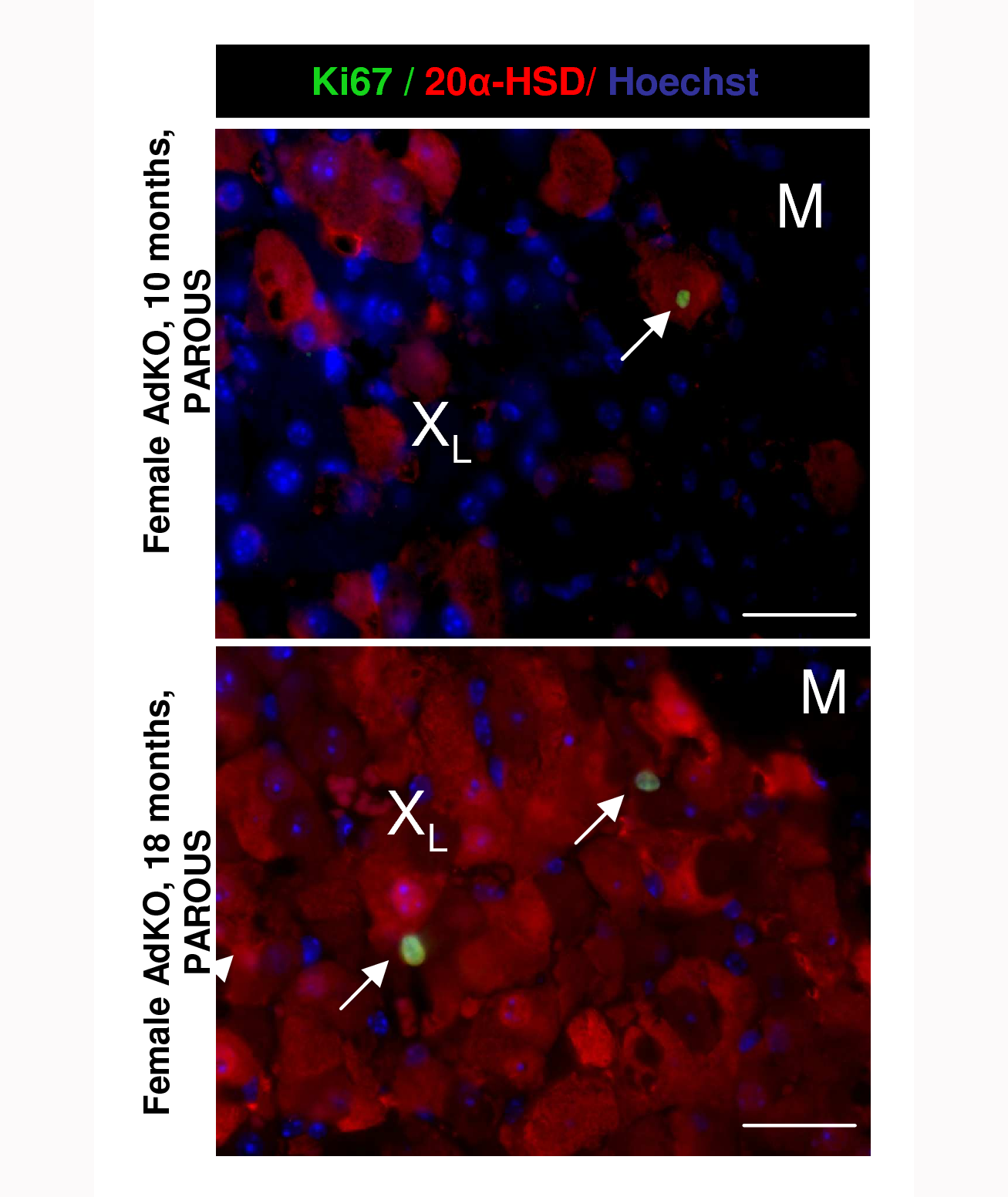

Supplement: Figure S8 — Evidence for cell proliferation in the X-like-zone of AdKO mice adrenals. In 10 and 18-month-old parous AdKO females, the X-zone 20α-HSD marker (in red) and the Ki67 proliferation marker (in green) were co-immunodetected and merged with the Hoechst nuclei marker (blue). Double-stained cells are outlined by arrows. XL, X-like-zone; Scale bars, 20 µm. (1.13 MB TIF) [file pgen.1000980.s008.tif]

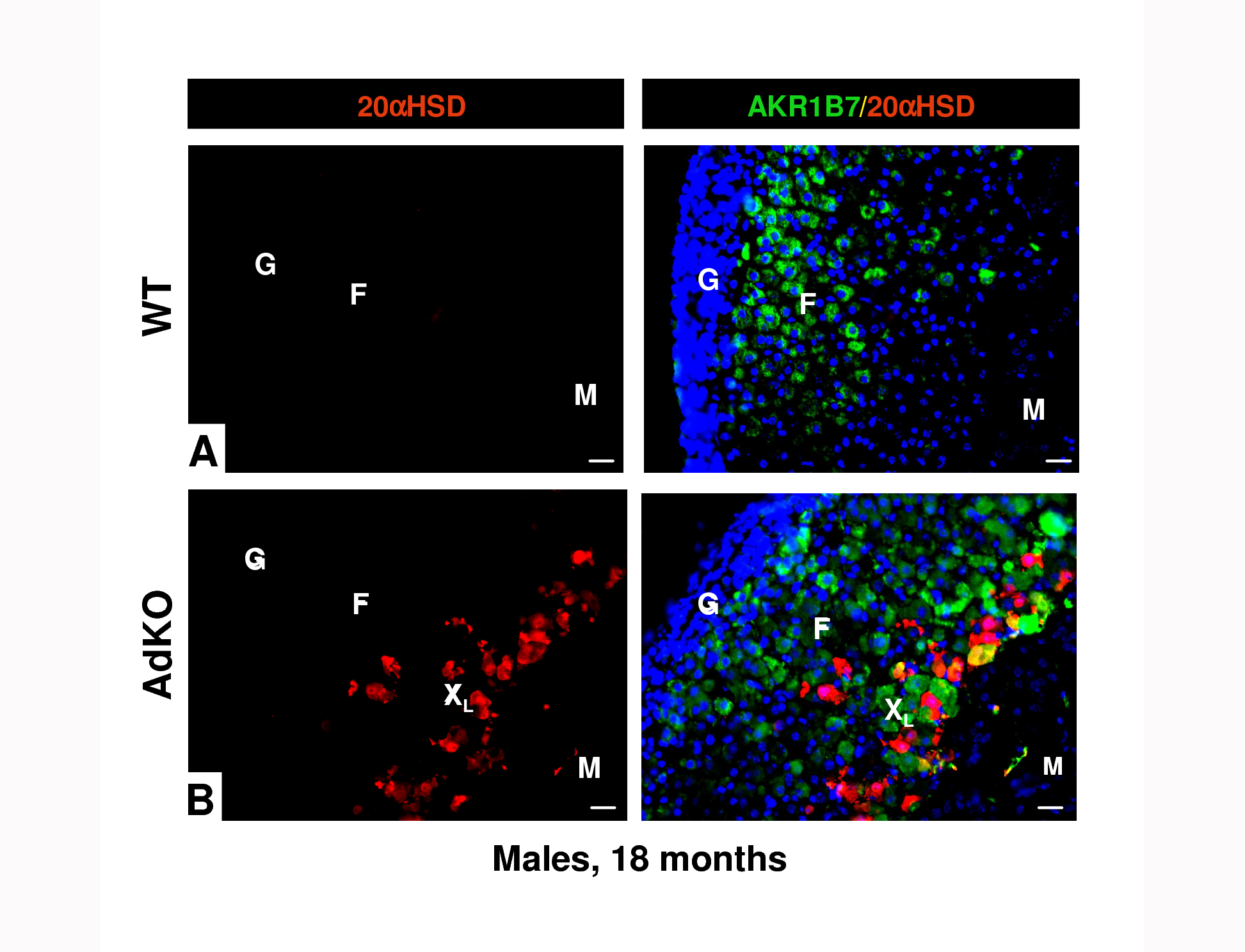

Supplement: Figure S9 — Existence of a persistent, mislocated X-like-zone in male AdKO adrenals. The X-zone 20α-HSD marker (in red) and the zona fasciculata Akr1b7 marker (green, right column) were immunodetected. The two colours are merged in the right column with the Hoechst nuclei marker (blue). A, Adrenal section of a 18-month-old WT male. No staining for 20α-HSD was shown. B, Adrenal section of an 18 month-old AdKO male. Cells doubled-stained for Akr1b7 and 20α-HSD were detected, as in AdKO females, indicating the presence of a pathological X-like-zone in AdKO male adrenals. M, Medulla; F, zona fasciculata; G, zona glomerulosa; X, X-zone; XL, X-like-zone; Scale bars, 20 µm. (1.37 MB TIF) [file pgen.1000980.s009.tif]

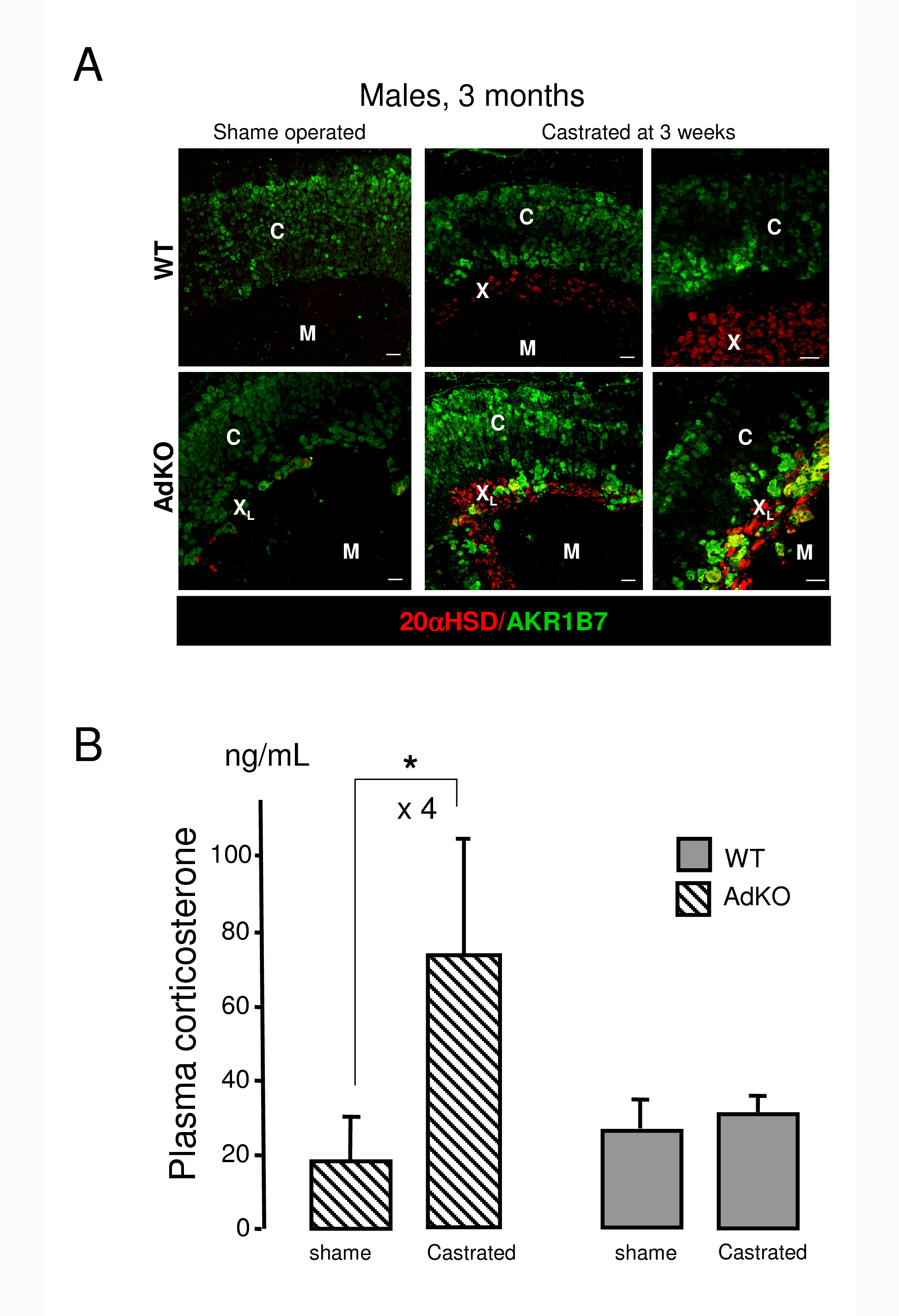

Supplement: Figure S10 — Castration in AdKO males increased the size of persistent, mislocated X-like-zone and plasma corticosterone levels. WT and AdKO males were castrated at 3 weeks and kept for sacrifice at 3 months of age. A, Co-immunostaining for Akr1b7 (green) and 20α-HSD (red) were realised on adrenal sections of control (left column) and castrated mice of WT and AdKO genotypes. B, Quantitative analysis of plasma corticosterone in control and castrated 3-month-old males of WT and AdKO genotypes. M, Medulla; C, cortex; X, X-zone; XL, X-like-zone; Scale bars, 20 µm. *, P<0.05. (1.91 MB TIF) [file pgen.1000980.s010.tif]

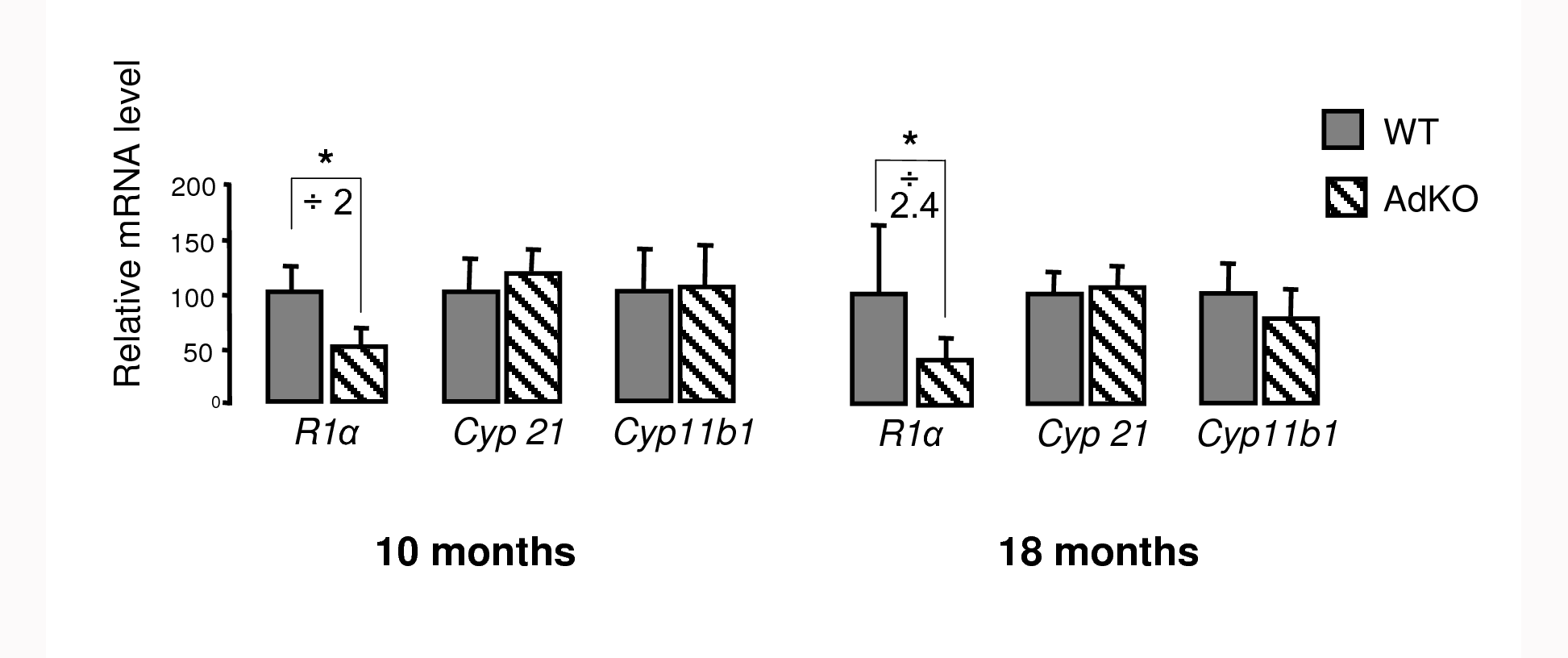

Supplement: Figure S11 — Maintenance of steroidogenesis markers in aging AdKO adrenals. Quantitative representation of mRNA levels of the genes: Prkar1a (control), Cyp21, Cyp11b1. RT-QPCRs were done with adrenal mRNA from WT and AdKO mice in 10 and 18-month-old females (parous). *, P<0.05. NS: statistically non significant. (0.09 MB TIF) [file pgen.1000980.s011.tif]
